# Sex‐specific differences in mortality and neurocardiac interactions in the Kv1.1 knockout mouse model of sudden unexpected death in epilepsy (SUDEP)

**DOI:** 10.1113/JP287582

**Published:** 2025-01-08

**Authors:** Kelsey Paulhus, Praveen Kumar, Kelly Kneale, T. Noah Hutson, Nicole M. Gautier‐Hall, Deng‐Shan Shiau, Megan Watts, Krystle Trosclair, Hemangini A. Dhaibar, Paari Dominic, Leonidas Iasemidis, Edward Glasscock

**Affiliations:** ^1^ Department of Biological Sciences Southern Methodist University Dallas TX USA; ^2^ Departments of Translational Neuroscience and Neurology Barrow Neurological Institute Phoenix AZ USA; ^3^ Department of Cellular Biology and Anatomy Louisiana State University Health Sciences Center Shreveport LA USA; ^4^ Department of Pharmacology, Toxicology & Neuroscience Louisiana State University Health Sciences Center Shreveport LA USA; ^5^ EpiFocus LLC Scottsdale AZ USA; ^6^ Department of Internal Medicine, Section of Cardiology Louisiana State University Health Sciences Center Shreveport LA USA

**Keywords:** brain‐heart interactions, epilepsy, Kv1.1, sex differences, SUDEP

## Abstract

**Abstract:**

Sudden unexpected death in epilepsy (SUDEP) is a devastating complication of epilepsy with possible sex‐specific risk factors, although the exact relationship between sex and SUDEP remains unclear. To investigate this, we studied *Kcna1* knockout (*Kcna1* ^−/−^) mice, which lack voltage‐gated Kv1.1 channel subunits and are widely used as a SUDEP model that mirrors key features in humans. To assess sex differences, we first performed survival analysis, EEG‐ECG recordings, seizure threshold testing and retrospective analysis of previous intracardiac pacing data. We then applied a novel modelling approach across organs (organomics) to uncover potential sex‐specific differences in brain–heart communication. Our findings revealed female *Kcna1* ^−/−^ mice have significantly longer lifespans than males, suggesting lower SUDEP rates. Although no sex differences were found in seizure frequency, duration, burden, susceptibility or interictal heart rate variability, females showed a higher incidence of bradycardia during spontaneous seizures than males, as well as resistance to inducible ventricular tachyarrhythmias in response to programmed electrical stimulation. Two captured SUDEP events, one per sex, displayed similar patterns of ictal bradycardia in both sexes, progressing to postictal cardiorespiratory failure. Going beyond traditional seizure and cardiac metrics, organomics analysis revealed that seizures affect brain–heart communication differently between sexes. Females exhibited more effective resetting of brain–heart interactions postictally than males. This finding may contribute to the lower SUDEP risk in females and underscores the complex interplay between sex, cardiac function and brain–heart communication in determining SUDEP susceptibility. Furthermore, seizure‐resetting measures could represent a promising class of biomarkers for SUDEP risk stratification.

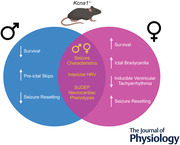

**Key points:**

- Female *Kcna1* ^−/−^ mice live longer than males, suggesting lower sudden unexpected death in epilepsy (SUDEP) rates.
- There are no sex differences in seizure metrics or interictal heart rate variability.
- Females show more bradycardia during seizures and are resistant to inducible ventricular tachyarrhythmias.
- Seizures affect brain–heart communication differently between the sexes.
- Seizures in females reset brain–heart interactions more effectively postictally, potentially lowering SUDEP risk.

## Graphical abstract

**Abstract figure legend** The *Kcna1* knockout mouse model of sudden unexpected death in epilepsy (SUDEP) exhibits sex‐specific differences in SUDEP risk. Female mice exhibit a lower SUDEP risk than males, despite similar seizure characteristics and interictal cardiac function across sexes. During seizures, females display a higher incidence of bradycardia and demonstrate resistance to inducible ventricular tachyarrhythmias. Organomics analysis indicates that females have more effective resetting of brain–heart interactions in the postictal period, suggesting a protective mechanism that may contribute to their reduced SUDEP susceptibility. During a SUDEP event captured for one male and one female, the terminal neurocardiac events were similar between the sexes. These findings highlight the importance of sex‐specific factors in SUDEP risk and brain–heart communication.

## Introduction

Epilepsy, a chronic neurological disorder characterized by recurrent spontaneous seizures, exhibits notable sex differences in its prevalence, manifestation, treatment outcomes and comorbidities. Although the incidence of epilepsy tends to be slightly higher in males, hormonal influences related to menstrual cycle and pregnancy can lead to increased seizure activity in females (Li et al., 2024; Shahla et al., 2018; Velíšková & DeSantis, 2013). Seizure types also tend to vary by sex, with males more probably experiencing generalized tonic‐clonic seizures (GTCS) and females more probably experiencing absence seizures (Hauser & Kurland, 1975; Kishk et al., 2019; Videira et al., 2021). Additionally, sex‐specific differences in drug metabolism related to hormones can affect anti‐seizure drug responsiveness (Giuliano et al., 2024). Epilepsy comorbidities show sex‐specific features as well, with women more probably experiencing depression and anxiety, whereas men have a higher risk of cognitive impairments (Berenbaum et al., 1997; Berger et al., 2018, 2017; Chan et al., 2015; Christian et al., 2020; Helmstaedter, 1999; Helmstaedter et al., 2004). These differences highlight the importance of considering sex as a factor in epilepsy treatment and management.

One catastrophic comorbidity of epilepsy that may be influenced by sex is a sudden unexpected death in epilepsy (SUDEP). SUDEP is defined as the sudden, unexpected death of a person with epilepsy, who was otherwise reasonably healthy and for which no obvious anatomical or toxicological cause of death is found upon postmortem examination (Nashef et al., 2012; Sveinsson et al., 2020). The exact underlying causes of SUDEP are still unknown, but it is generally believed to result from seizure‐related cardiorespiratory failure, typically occurring in the minutes to hours following a seizure, that is, during the postictal period (Devinsky, 2011). Several SUDEP risk factors have been identified, including the presence of any GTCS, GTCS occurring more than three times per year and lack of seizure freedom in the last 1–5 years (Harden et al., 2016). Although some epidemiological studies have associated being male with increased SUDEP risk, others have failed to find a link between sex and SUDEP risk (Devinsky et al., 2016; McHugh & Delanty, 2008; Nilsson et al., 1999; Tarighati Rasekhi et al., 2021), drawing into question whether sex influences SUDEP susceptibility.

In the present study, we investigated sex differences in SUDEP risk in *Kcna1* knockout (*Kcna1^−/−^*) mice, a widely used model of SUDEP. In our previous studies with this animal model, we have observed that males tend to die earlier than females, but we never conducted a systematic survival and electrophysiological study to validate this potential sex difference. The *Kcna1* gene encodes pore‐forming Kv1.1 voltage‐gated potassium channel α‐subunits that are expressed in both the brain and heart, where they control action potential firing properties (Glasscock et al., 2015; Jan & Jan, 2012; Wang et al., 1993, 1994). In humans, more than 20 mutations in *KCNA1 h*ave been linked to epilepsy (Paulhus & Glasscock, 2023). Mice lacking *Kcna1* exhibit hallmark features of human SUDEP, including frequent GTCS and seizure‐related sudden death involving cardiorespiratory dysfunction (Dhaibar et al., 2019; Glasscock et al., 2010; Smart et al., 1998). These mice also exhibit evidence of impaired brain–heart interactions which could underlie their elevated SUDEP risk, such as decreased brain–heart association and connectivity, increased parasympathetic‐mediated cardiac conduction blocks and increased survival in response to vagotomy (Glasscock et al., 2010; Hutson et al., 2020; Mishra et al., 2017; Moore et al., 2014).

As a first step, in the present study, we re‐examined our mouse survival logs and verified that males indeed exhibit significantly higher premature mortality than females. Using simultaneous video‐EEG‐ECG recordings and seizure threshold tests, we then investigated whether sex differences in seizure and cardiac phenotypes of *Kcna1^−/−^* mice could explain their different SUDEP rates. We also retrospectively mined intracardiac pacing data from a previous study (Trosclair et al., 2021) aiming to identify potential sex differences in ventricular arrhythmia susceptibility. We next applied a novel organomics approach to detect peri‐ictal differences in the patterns of brain–heart communication in males *vs*. females. Although traditional EEG‐ECG measures revealed only minimal sex differences, the organomics analysis showed that seizures influence brain–heart interactions differently between males and females. In particular, the organomics analysis showed that female *Kcna1^−/−^* mice may experience less SUDEP because their seizures produce more effective neurocardiac resetting postictally, which could provide a protective homeostatic mechanism against cardiorespiratory failure.

## Methods

### Ethical approval

The investigators understand the ethical principles under which the *Journal of Physiology* operates, and ensure the work presented here complies with their animal ethics checklist. All experiments were performed on animals following National Institutes of Health (NIH) guidelines with approval from the Institutional Animal Care and Use Committee of Louisiana State University Health Science Centre (LSUHSC), Shreveport, LA. The approved protocol number for these experiments was P‐16‐021.

### Animals and genotyping

*Kcna1^−/−^* mice (Tac:N:NIHS‐BC genetic background) carry null (knockout; KO) alleles of the *Kcna1* gene generated by targeted deletion of the entire open reading frame on chromosome 6 (Smart et al., 1998). The mice were originally obtained from the lab of Dr. Philip Schwartzkroin in the Department of Neurological Surgery in the School of Medicine at the University of California‐Davis (Sacramento, CA, USA). Western blot analysis and immunohistochemical analysis have previously confirmed the deletion of Kv1.1 protein in this strain of *Kcna1^−/−^* mouse (Glasscock et al., 2012, 2010; Smart et al., 1998). Mice were housed in standard cages with bedding material, maintained at a temperature of ∼22°C. They had *ad libitum* access to food and water and were maintained under a 12:12 h light/dark photocycle. For genotyping, genomic DNA was isolated by enzymatic digestion of tail clips using Direct‐PCR Lysis Reagent (Viagen Biotech, Los Angeles, CA, USA). Genotypes were determined by performing PCR amplification of genomic DNA using allele‐specific primers. The following primer sequences were used to yield genotype‐specific amplicons of ∼337 bp for the wild‐type (WT) allele and ∼475 bp for the KO allele: a WT‐specific primer (5′‐GCCTCTGACAGTGACCTCAGC‐3′), a KO‐specific primer (5′‐CTTCTATCGCCTTCTTGACG‐3′) and a common primer (5′‐GCTTCAGGTTCGCCACTCCCC‐3′). (Smart et al., 1998) The lifespans of all KO mice in our colony were logged as a standard lab protocol and used to generate the male and female survival curves. The lifespan data have been reported previously without being separated by sex (Trosclair et al., 2020); however, in the present study, the data were reanalysed and the difference between males and females was measured and reported. Upon completion of experiments, animals that did not die prematurely were humanely killed by isoflurane overdose (Aspen Veterinary Resources, Liberty, MO, USA) and subsequent decapitation.

### Simultaneous EEG‐ECG recordings

Mice of both sexes (postnatal days 29–31) were anaesthetized using an anaesthetic cocktail and surgically implanted with bilateral silver wire electrodes (0.005 inch in diameter) connected to a microminiature connector (Omnetics Connector Corporation, Minneapolis, MN, USA) for recording in a tethered configuration. The anaesthetic cocktail contained ketamine (80–100 mg kg^−1^), xylazine (6–10 mg kg^−1^) and acepromazine (1–2 mg kg^−1^), and was administered by i.p. injection. Sufficient depth of anaesthesia was confirmed by a lack of tail or toe pinch reflex and was monitored throughout each surgery. EEG electrodes were inserted into the subdural space through cranial burr holes overlying the parietotemporal cortex for recording electrodes and above the frontal cortex for reference and ground electrodes. For ECG, two thoracic electrodes were tunnelled s.c. on either side and sutured in place to record cardiac activity. Carprofen injections (5 mg kg^−1^, s.c.) were given at the end of the surgery and 24 h later to minimize pain. Mice were allowed to recover for 48 h before recording 31–60 h of continuous EEG‐ECG activity using a digital video monitoring system (Data Sciences International, St Paul, MN, USA). Signals were sampled at 1 kHz for EEG and 2 kHz for ECG.

The EEG and ECG biosignals were analysed offline to identify spontaneous seizures and cardiac events using Ponemah software (Data Sciences International). The following digital filter settings were used for offline analysis unless otherwise stated: a 75 Hz low‐pass and 0.3 Hz high‐pass filter for EEG and a 3 Hz high‐pass filter for ECG. Seizures were identified by visual inspection of the EEG and defined as high‐amplitude (at least two times the baseline), rhythmic electrographic discharges lasting at least 5 s. Seizure frequency was calculated as the total number of seizures in the recording divided by the total number of recording hours for each individual animal and expressed as seizures h^−1^. Seizure duration was defined as the time from the onset of electrographic seizure (typically a large high amplitude spike followed by sudden voltage depression) until the cessation of spiking. Seizure burden was calculated from the total seconds of seizure activity in the recording divided by the total number of recording hours and represented as seconds seizing per hour. Behavioural seizure severity was manually classified from video footage and scored using a modified Racine scale as reported previously (Mishra et al., 2017): stage 1, myoclonic jerk; stage 2, head nodding/stereotypy; stage 3, forelimb/ hind limb clonus, tail extension, a single rearing event; stage 4, continuous rearing and falling; and stage 5, severe tonic–clonic convulsions.

### Heart rate (HR) and heart rate variability (HRV) analyses

Quantification of cardiac features was performed using Ponemah software (Data Sciences International) as reported previously (Dhaibar et al., 2019; Trosclair et al., 2020). Briefly, for estimating HR and HRV, six separate RR interval series were derived by sampling 2 min ECG segments every 4 h to provide three light phase (06.00 h to 18.00 h) and three dark phase (18.00 h to 06.00 h) measurements. Measurements were taken from the first 24 h of recording. The HR and HRV values for each animal were then averaged from the six total segments. RR intervals were only sampled during times when the mouse was stationary and when the ECG showed no abnormalities, such as skipped or ectopic beats. Skipped beats (e.g. cardiac conduction blocks or sinus pauses) were identified from the ECG waveforms and defined as a prolongation of the RR interval equalling ≥1.5 times the previous RR interval. For cardiac measures related to rate, bradycardia was defined as a rate change of at least −20% relative to the 20 s pre‐ictal period immediately before seizure onset. Pre‐ and postictal phenotypes were counted if they occurred in the 20 s before seizure onset or after its termination, respectively.

### Flurothyl‐induced seizure susceptibility

Flurothyl‐induced seizure susceptibility was performed using the same procedure as reported previously (Vanhoof‐Villalba et al., 2018). Mice (postnatal days 29–31) were placed in an air‐tight Plexiglas chamber (18.4 × 15.0 × 34.5 cm) and allowed to acclimate for 5 min before being exposed to the liquid convulsant flurothyl (2,2,2‐trifluoroethyl ether; Sigma‐Aldrich, St Louis, MO, USA). Flurothyl was infused (20 µL min^−1^) with a syringe pump onto Whatman filter paper (Cytiva, Marlborough, MA, USA) suspended at the top of the chamber from which it was vaporized. The latency (in seconds) was measured as the time from the first drip of flurothyl to the onset of a fully generalized (tonic‐clonic) seizure, which typically consisted of ∼3–5 s of falling and/or running/bouncing. Immediately following generalized seizure onset, the mice were quickly removed to fresh air and placed in a cage for observation and recovery. Each mouse was tested individually and received only one exposure to flurothyl. The testing chamber was cleaned and aerated in between animals.

### Intracardiac electrophysiology

*In vivo* pacing studies of 4‐month‐old KO and WT mice (Trosclair et al., 2021) were retrospectively analysed for sex differences. In these studies, mice were anaesthetized with isoflurane and placed supine on a temperature‐controlled platform with limbs taped onto ECG electrodes. An octapolar electrode catheter was then surgically inserted into the internal right jugular vein and advanced to the right atrium and ventricle to administer electrical stimulation and record intracardiac electrograms. To induce ventricular tachyarrhythmias (VTA), programmed electrical stimulation (PES) was performed using 2 ms current pulses at 400 µA delivered by an external stimulator. The protocol included burst pacing sequences repeated 20 times over 70.4 s. VTA was defined as rapid spontaneous ventricular depolarizations lasting >500 ms. Each mouse underwent three PES trials and was considered VTA‐positive if VTA occurred in any trial. After baseline trials, the β‐adrenergic receptor agonist isoproterenol hydrochloride was injected (4 mg kg^−1^, i.p.) to increase arrhythmia susceptibility and PES was repeated. All VTAs were self‐terminating.

### Estimation of brain–heart connectivity

Generalized partial directed coherence (GPDC) was used as a measure of frequency‐dependent directed and normalized connections between the brain (left and right EEG) and heart (ECG) in male and female animals. For the estimation of GPDC, the data were fit to a 3‐D (two‐channel EEG and one‐channel ECG) multivariate autoregressive (MVAR) model (order of 7) for consecutive and non‐overlapping 10 s segments from each recording period per animal. The segment's size was selected as large enough to capture multiple cycles of potentially relevant physiological patterns and robust MVAR estimation. The MVAR coefficients were then used in the estimation of GPDC values in the frequency domain, which were finally averaged over 10 Hz frequency bands ranging from 1–200 Hz, as reported previously (Baccalá et al., 2013; Hutson et al., 2020). The decision to analyse data between 1 and 200 Hz was based on the Nyquist theorem. With a sampling rate of 1 kHz, the theorem suggests an upper limit of one‐half the sampling rate, or one‐third in common practice, which corresponds to frequencies up to 333 Hz in our data without aliasing. To be conservative, an upper limit of 200 Hz was applied for our calculations. Thus, directional interactions were aggregated so that one value of brain → heart and heart → brain connectivity was generated per 10 s window and 10 Hz frequency band. This analysis resulted in frequency‐dependent (10 Hz resolution) brain–heart connectivity profiles over time (10 s resolution) for the full EEG‐ECG records of all animals.

### Seizure resetting index (SRI)

To quantitatively evaluate the seizure‐induced change from the preictal to postictal states, equally sized (5 min) preictal (prior to seizure onset) and post‐ictal (following seizure offset) windows were defined. Postictal and preictal connectivities, estimated by the GDPC values per direction (brain → heart and heart → brain) every 10 s, were compared using the *T*‐index, that is, the two‐sample *t* test value for the mean, by estimating the difference between the means of connectivity during the peri‐ictal periods (i.e. postictal minus preictal) divided by their joint SD:

$$T-\mathrm{index}\;\left(f,i\right)=\frac{\mu_{2}\left(f,i\right)-\mu_{1}\left(f,i\right)}{\sqrt{\frac{{\sigma_{1}}^{2}\left(f,i\right)}{n_{1}}+\frac{{\sigma_{2}}^{2}\left(f,i\right)}{n_{2}}}}$$

where $\mu_{1}\left(f,i\right)$ and $\mu_{2}\left(f,i\right)$ are the sample means of the GPDC values from the pre‐ictal and post‐ictal segments, respectively, at frequency *f* and seizure *i*, and per directional functional connection. The $\sigma_{1}\left(f,i\right)$ and $\sigma_{2}\left(f,i\right)$ are the respective sample (GPDC) SDs (and $\frac{\sigma_{1}}{\sqrt{n_{1}}}$ and $\frac{\sigma_{2}}{\sqrt{n_{2\;}}}$ are the respective SDs of the sample means $\mu_{1}\left(f,i\right)$ and $\mu_{2}\left(f,i\right)$). In our case, *n*_1_ = *n*_2_ = 30 because we use equally sized pre‐ictal and post‐ictal windows of 5 min, composed of 30 segments of 10 s. Consecutive seizures with overlap between their respective post‐ictal and subsequent pre‐ictal periods were excluded from the analysis. Because seizures tended to exhibit more clustering in males than females, more seizures were available for seizure resetting analysis in females (142 seizures) than males (55 seizures) (Table 1). The SRI per direction, sex and frequency (*f*) was estimated as the mean of *T*‐indices across all seizures in the female or male group per direction:

$$SRI\;\left(f\right)=\frac{1}{N}\;\sum_{i=1}^{N}T-\mathrm{index}\;\left(f,i\right)$$

**Table 1:** Seizures per female (F) and male (M) mouse used for organomics analysis

Female		Male	
Mouse ID	Number of seizures	Mouse ID	Number of seizures
F1	6	M1	14
F2	3	M2	4
F3	32	M3	20
F4	44	M4	5
F5	57	M5	1
		M6	11
**Total**	**142**	**Total**	**55**

The SD of SRI per sex, as well as the SD of [female–male] sex difference and its *P* value, were estimated from empirical distributions created by 10,000 new mean *T*‐index values, each one from a new dataset of mice, by sampling and random replacement of the original mice, and accompanied by their respective seizures (for more details, see the section below titled “Statistical analysis of resetting”). An individual seizure was considered to reset if its *T*‐index exceeded the threshold (*T*_th_) of 0.5273, which corresponds to the 70th percentile of the two‐sample *t* distribution with (30 + 30 – 2 = ) 58 degrees of freedom.

To investigate whether SRI could be used to predict the sex of a mouse, linear discriminant analysis (LDA) was performed using R software (https://cran.r‐project.org/doc/manuals/r‐release/R‐intro.html), with SRI values as predictors and the mouse's sex as the response variable. The SRI values included were one of the low and one of the high‐frequency bands in the heart → brain interaction (1–10 Hz and 100–110 Hz, respectively) and one of the high‐frequency bands in the brain → heart interaction (80–90 Hz), chosen based on their highest significant sex differences (positive or negative) in mean SRI (Fig. 4). Leave‐one‐out cross‐validation was then applied to assess the prediction accuracy. The accuracy of the model for each sex was quantified using:

$$\begin{array}{ccc} \mathrm{Accuracy} & = & \left(\mathrm{Sensitivit}y^{F}\times N^{F}+\mathrm{Specificit}y^{F}\right.\\ & & \left.\times \;N^{M}\right)/\left(N^{F}+N^{M}\right) \end{array}$$

$$\begin{array}{ccc} \mathrm{or} & = & \left(\mathrm{Sensitivit}y^{M}\times N^{M}+\mathrm{Specificit}y^{M}\right.\\ & & \left.\times \;N^{F}\right)/\left(N^{F}+N^{M}\right) \end{array}$$

where *N* is the sample size of females (F) or males (M); sensitivity is a percentage of true positive; and specificity is a percentage of true negative.

### Proportion of resetting seizures

The proportion of resetting seizures (PRS) per sex was calculated as the number of resetting seizures divided by the total number of seizures. To estimate the SD of the PRS, an empirical distribution of 10,000 new PRS values was created by generating new datasets of mice and their seizures via resampling and replacement of the original mice and their own seizures. After calculating the sex difference in PRS, the SD and *P* value for this difference were estimated using analogously created empirical distributions from 10,000 new [PRS(female) – PRS(male)] values (for more details, see the section below titled “Statistical analysis of resetting”).

### Statistical analyses and blinding

Analysis of biosignal recordings was performed in a blinded fashion using file names that obscured genotype and sex. All data are expressed as the mean ± SD unless otherwise stated. Prism, version 10 (GraphPad Software Inc., La Jolla, CA, USA) was used for statistical analysis. Normality was assessed prior to statistical analysis. Survival curves were evaluated using the Kaplan–Meier log‐rank (Mantel–Cox) test. For most comparisons involving only two groups, unpaired two‐tailed Student's *t* tests were employed as specified. However, the seizure clustering data was not normally distributed, and so the non‐parametric Mann–Whitney test was used. Furthermore, for comparing seizure resetting between males and females, new probability distributions were created using R software, version 4.2.1; for details on how the empirical distributions were constructed, see below). This approach accounted for the likelihood that seizures within the same animal are not independent. We then assessed statistical significance using the bootstrap method, a non‐parametric resampling technique (Efron & Tibshirani, 1994; Kelly et al., 2010). This method is recommended when samples lack independence, but existing data correlations need to be preserved (for more details, see the section below titled “Statistical analysis of resetting”). For comparisons involving three or more groups, one‐way ANOVA was performed with Tukey's multiple comparison *post hoc* tests. Intracardiac electrophysiology was analysed using a two‐tailed Fisher's exact test. Outliers were identified using the ROUT method with *Q* = 1%, resulting in one female data point being excluded from the SD of the beat‐to‐beat intervals (SDNN) analysis. *P* < 0.05 was considered statistically significant for differences between groups.

#### Statistical analysis of resetting

To test the hypothesis that females have more effective seizure resetting than males, we created new probability distributions by employing R software, version 4.2.1. The reason is that seizures within the same subject are more probably correlated than independent from each other (Iasemidis et al., 1994). This could be a result of the existence of the same mechanism of ictogenesis in the same subject, as well as the dependence of a seizure occurrence upon the resetting power of the previous seizure, a hypothesis that could also explain seizure clustering. Therefore, in our estimation of statistics (mean ± SD, *P* value) of different measures (*T*‐index and PRS) within and across the two groups of subjects (male and females), we needed to take into consideration that seizures in the same subject may not be independent, and hence non‐parametric tests, such as the Wilcoxon rank‐sum test (Mann–Whitney *U* test) that works for independent samples only, are problematic. The non‐parametric test that is recommended in this situation is the construction of the empirical probability distribution via the bootstrap (resampling) method that preserves any existing correlations in the data. The SD and all *P* values in Fig. 4 and Tables 2 and 3 were estimated by the appropriate application of this method.

The bootstrap method samples (and may replace) a subject at a time from the original dataset of the *N* subjects, repeats this sampling *N* times randomly, and creates a new dataset of subjects, each subject with their own original seizures, so that any existing seizure dependency is maintained in the new dataset of seizures. One resampling example (Table 1) could be:

Female: F2, F3, F4, F4, F5, so there would be 3 + 32 + 44 + 44 + 57 = 180 seizures
Male: M2, M2, M3, M3, M5, M6, so there would be 4 + 4 + 20 + 20 + 1 + 11 = 60 seizures

The above creation of a new dataset was repeated 10,000 times with new male and female datasets and with each one with their own seizures, and from each dataset the measures of mean *T*‐index and PRS were estimated (per sex). The SD is estimated from the resulting empirical distribution of the new 10,000 mean *T*‐index and PRS values, respectively.

If we now want to test a hypothesis with a *P* value, the bootstrap procedure changes slightly. If *N*_M_ and *N*_F_ are the numbers of males and females in our original groups of mice, the null hypothesis is that both sexes belong to the same population (that is, they do not differ) with respect to *T*‐index and PRS and so, by creating two groups randomly, their *T*‐index and PRS values should not be statistically different from those in the original groups. In this case, the two groups (*) are recreated 10,000 times with *N*_M_ and *N*_F_ numbers of subjects and their own seizures, by resampling and replacing one at a time from the combined population of *N*_M_ + *N*_F_ mice. One resampling example (Table 1) here could be:

Female*: F2, M2, M1, F4, F5, so there would be 3 + 4 + 14 + 44 + 57 = 122 seizures
Male*: M2, F4, M3, M3, M5, F5, so there would be 4 + 44 + 20 + 20 + 1 + 57 = 146 seizures

Then, the empirical distributions of the two statistics [difference of mean (*T*‐indices) and difference of PRSs between males and females] were estimated, and the *P* values for the hypotheses testing were obtained based on the location of the observed statistics in the created null distributions.

1. **PRS and its SD per sex**, where a seizure was considered resetting when the *T*‐index [post mean GPDC – pre mean GPDC] between 5 min preictal (30 GPDC values) and 5 min postictal (30 GPDC values) is larger than the *T*‐index threshold (*T*_th_) that corresponds to 70% percentile of the two‐sample t‐distribution with 30 + 30 − 2 = 58 degrees of freedom, that is, *T*_th = _0.5273. Then, PRS = (# resetting seizures/total # seizures) per sex. The SD of the PRS was estimated from the empirical distribution of PRS that was created from 10,000 new PRS values; each new PRS was estimated from each new dataset of seizures (mice and their seizures) that was generated by resampling/replacement of the original mice and their own seizures. The SD and *P* value for (F–M) were estimated from analogously created empirical distributions from 10,000 new [PRS(F)–PRS(M)] values.
2. **SRI**: Mean *T*‐index (postictal–preictal) and its SD across seizures per sex. The mean *T*‐index (post–pre) per sex was estimated as the [sum of all *T*‐indices (post–pre) across seizures/total # seizures]. The SD per sex, as well as the SD of F–M and the *P* value for the F–M across sexes, were estimated from empirical distributions created by 10,000 new mean *T*‐index values.

## Results

### Male *Kcna1^–/–^* mice have higher SUDEP rates than females

**Figure 1:**
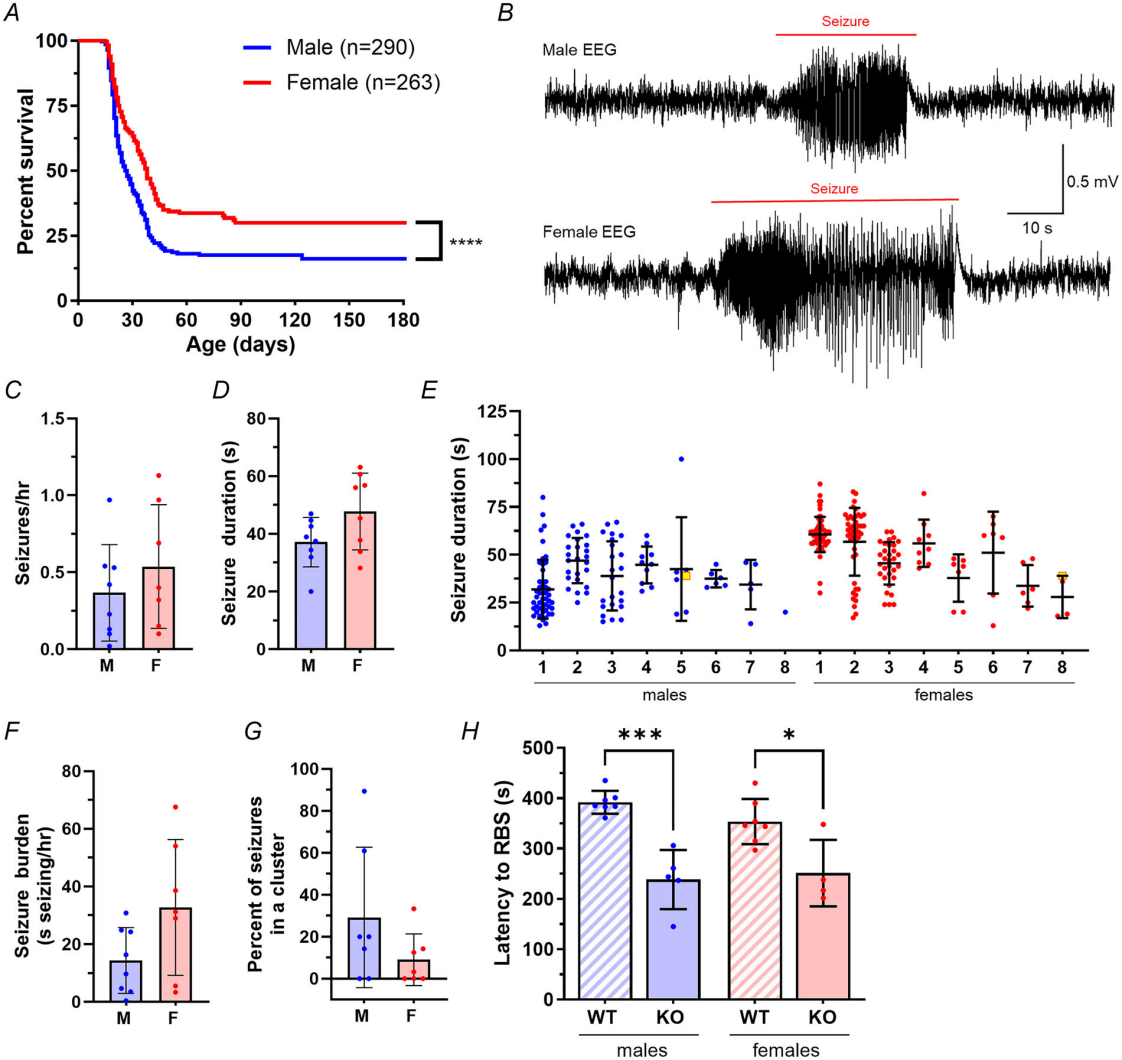
Male *Kcna1* ^–/–^ mice show enhanced mortality without major differences in EEG characteristics or seizure threshold *A*, Kaplan–Meier survival curves showing lifespan for male (*n* = 290) and female (*n* = 263) *Kcna1* ^–/–^ mice. In male *Kcna1* ^–/–^ mice, survival was significantly decreased compared to female *Kcna1* ^–/–^ mice (*P* = 0.000002, log‐rank test). *B*, representative EEG traces from a male and female *Kcna1* ^–/–^ mouse showing similar qualitative seizure characteristics. Quantification of seizure frequency (*P = *0.375) (*C*) and seizure duration (*P = *0.079) (*D*) for male (*n* = 8) and female (*n* = 7–8) *Kcna1* ^–/–^ mice. *E*, plot of individual seizure durations for every seizure recorded for male (*n* = 8) and female (*n* = 8) *Kcna1* ^–/–^ mice. Male mouse #5 and female mouse #8 died from SUDEP during recording, and their terminal seizures are indicated by yellow squares with orange outlines. *F*, quantification of seizure burden (*P = *0.070) in male (*n* = 8) and female (*n* = 7) *Kcna1* ^–/–^ mice. *G*, quantification of the percentage of seizures occurring as part of a cluster (*P = *0.210), defined as occurring within 30 min of another seizure, in male (*n* = 7) and female (*n* = 7) *Kcna1* ^–/–^ mice. One female was excluded from analysis of seizure frequency, burden and clustering because it had an incomplete dataset as a result of SUDEP occurring early in the recording session. One male had a single seizure and therefore could not be included in the seizure clustering analysis. *H*, quantification of the latency to a generalized running and bouncing seizure (RBS) in response to flurothyl exposure as a measure of seizure threshold. Male (*n* = 5) and female (*n* = 4) *Kcna1* ^–/–^ mice exhibited significantly reduced latencies (one‐way ANOVA, *P = *0.00004) compared to their male (*n* = 7) and female (*n* = 7) WT counterparts (*post hoc* Tukey's multiple comparison test: WT male *vs*. KO male, *P = *0.0001; WT female *vs*. KO female, *P = *0.012). F, female; KO, knockout; M, male; WT, wildtype. ***P < 0.001 and *P < 0.05.

We have anecdotally observed that female *Kcna1^–/–^* mice experience less premature mortality as a result of SUDEP than their male counterparts, but we had never verified this by systematic quantification. Towards this goal, we retrospectively analysed several years of our mouse records to compare the ages at which natural death occurred for male and female *Kcna1^–/–^* mice. Importantly, we have not observed premature lethality in WT (*Kcna1^+/+^*) and heterozygous (*Kcna1^+/–^*) littermate mice. We found that male *Kcna1^–/–^* mice (*n* = 290) exhibited a median survival age of 26.5 days that was significantly shorter compared to females (*n* = 263) which exhibited a median survival of 38 days (*P = *0.000002) (Fig. 1*A*). In both sexes, there was a window of susceptibility to premature death, beginning at ∼15 days old and continuing until ∼60 days old, after which time premature death occurred only rarely and sporadically. About twice as many females (34%) survived to 60 days old compared to males (18%). *Kcna1^–/–^* mice have previously been observed to die occasionally from status epilepticus (SE; i.e. repeated seizures occurring within 5 min of one another without recovery in between) (Smart et al., 1998). However, we found the majority of deceased *Kcna1^–/–^* mice in an outstretched position suggesting tonic hindlimb extension indicative of death immediately following a GTCS and thereby consistent with SUDEP. Because most deaths (≥75%) occurred at early ages before the time of sexual maturity, which averages ∼40 days in mice, the contribution of circulating gonadal hormones should have been minimal (Gordon & Corbitt, 2015). Of note, because many aspects of these sudden death events in *Kcna1^–/–^* mice resemble SUDEP in humans, we refer to these premature deaths as ‘SUDEP’ in this study. However, we acknowledge that the mice may not model the full complexity of human SUDEP and that some deaths may result from SE.

### Traditional EEG seizure characteristics are not significantly different between the sexes

We hypothesized that one explanation for the increased SUDEP susceptibility in male *Kcna1^–/–^* mice could be that they have a more severe seizure disorder compared to females. Therefore, we performed video EEG‐ECG recordings in male (*n* = 8) and female (*n* = 8) mice to measure their electrographic and behavioural seizure characteristics. Electrographic seizure patterns appeared qualitatively similar in both sexes (Fig. 1*B*), and males and females showed comparable seizure frequencies (*P* = 0.375) (Fig. 1*C*). However, females tended to have longer average seizure durations (*P* = 0.079) (Fig. 1*D* and *E*) and higher seizure burdens than males (*P* = 0.070) (Fig. 1*F*). We next compared inter‐seizure intervals to identify potential sex differences in seizure clustering patterns which could be associated with adverse outcomes. In males, an average of 29% of seizures occurred within 30 min of another seizure compared to only 9% in females, although this difference was not statistically significant (*P* = 0.210) (Fig. 1*G*). We next aimed to evaluate potential sex differences in behavioural seizure severity using a modified Racine scale to score the behaviour associated with each seizure. Females tended to exhibit higher seizure scores on average (2.8 ± 0.1) compared to males (2.2 ± 0.3), indicative of more severe behavioural seizures, but again these differences were not statistically significant (*P* = 0.116). Thus, analysis of traditional EEG seizure metrics suggested that the increased mortality in males could not be attributed to a more severe seizure phenotype.

### *Kcna1^–/–^* mice exhibit no sex differences in seizure threshold

To test whether the sexes exhibit differences in susceptibility to inducible seizures, we exposed mice to the volatile liquid convulsant flurothyl and measured the latency to running‐bouncing generalized tonic–clonic seizures as an estimate of seizure threshold. Upon flurothyl exposure, both male (*n* = 5; *P* = 0.0001) and female *Kcna1^–/–^* mice (*n* = 4; *P* = 0.012) exhibited significantly reduced seizure latencies compared to WT (i.e. *Kcna1^+/+^*) controls of the same sex (*n* = 7 per sex), respectively (Fig. 1*H*). However, male and female *Kcna1^–/–^* mice exhibited similar seizure latencies suggesting no significant sex differences at the level of seizure threshold.

### *Kcna1^–/–^* mice exhibit sex differences in preictal and ictal cardiac dysfunction

**Figure 2:**
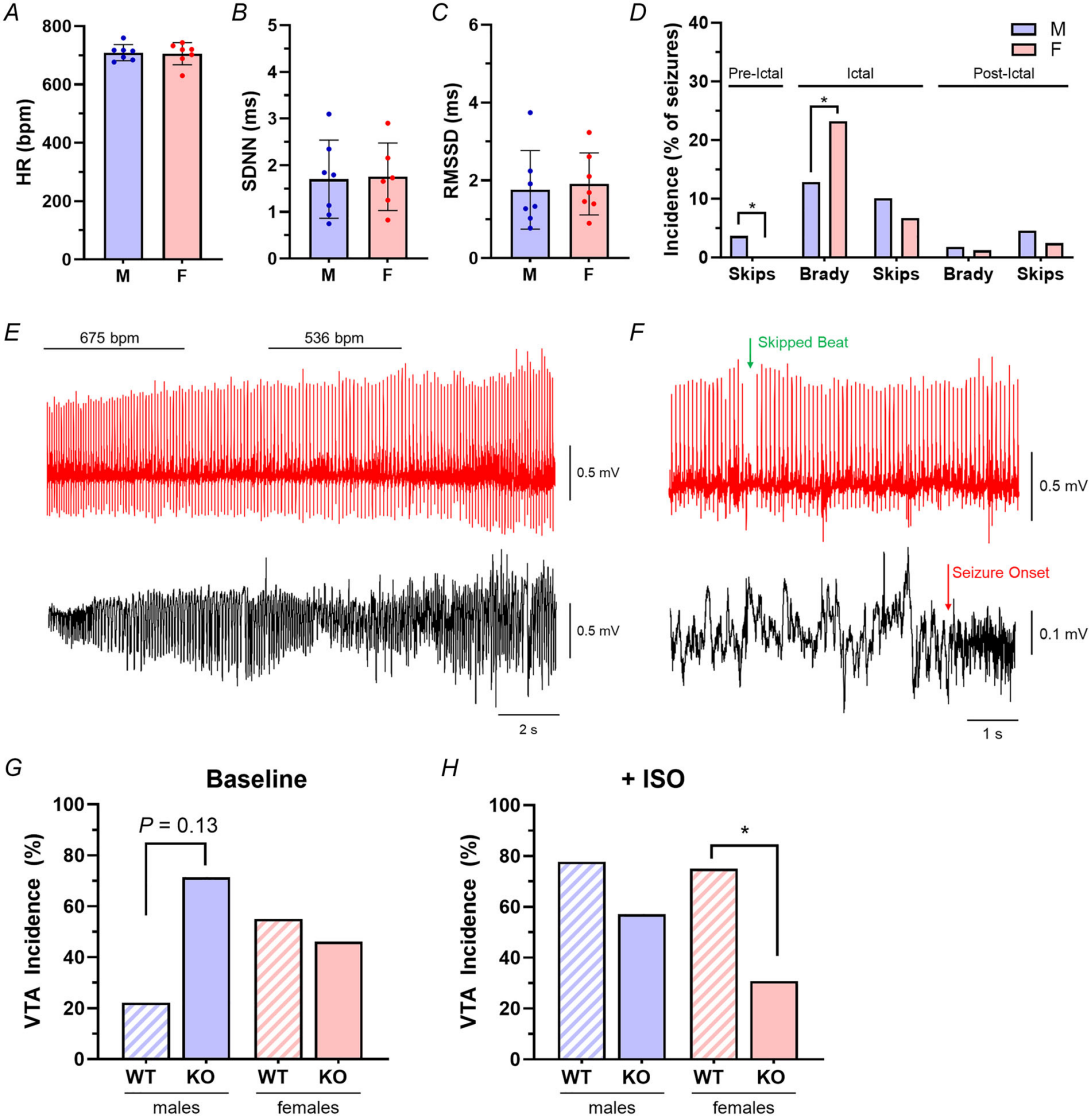
*Kcna1* ^–/–^ mice exhibit sex‐specific differences in seizure‐related cardiac dysfunction and intracardiac pacing‐induced arrhythmia susceptibility Quantification of interictal cardiac measures for HR (*P = *0.841) (*A*), SDNN as an estimate of total autonomic activity (*P = *0.907) (*B*) and RMSSD (*P = *0.756) (*C*) as an estimate of parasympathetic activity for male (*n* = 7) and female (*n* = 7) *Kcna1* ^–/–^ mice. One male was excluded from all cardiac analysis because of ECG artifacts which hindered accurate waveform analysis. One female was excluded from all cardiac analysis because of incomplete data as a result of SUDEP occurring early in the recording session. *D*, quantification of the incidence of various cardiac abnormalities during pre‐ictal, seizures (ictal) and post‐ictal periods, represented as the percentage of total seizures in which they occurred. Male *Kcna1* ^–/–^ mice had significantly more pre‐ictal blocks (*P = *0.025) and female *Kcna1* ^–/–^ mice had significantly more ictal bradycardia (*P = *0.041). There were 109 seizures analysed in eight males and 164 seizures analysed in seven females. In male mice, the ratio of specific cardiac events to total seizures was 4/109 for pre‐ictal skipped heartbeats, 14/109 for ictal bradycardia, 11/109 for ictal skipped heartbeats, 2/109 for post‐ictal bradycardia and 5/109 for post‐ictal skipped heartbeats. In female mice, the ratio of specific cardiac events to total seizures was 0/164 for pre‐ictal skipped heartbeats, 38/164 for ictal bradycardia, 11/164 for ictal skipped heartbeats, 2/164 for post‐ictal bradycardia and 4/164 for post‐ictal skipped heartbeats. *E*, representative ECG trace showing ictal bradycardia in a female *Kcna1* ^–/–^ mouse with corresponding EEG seizure activity. The heart rates before (left) and during (right) the bradycardia event are indicated by bars above the corresponding ECG segments with the rates labelled in beats per minute (bpm). *F*, representative ECG trace showing a pre‐ictal skipped heartbeat (green arrow) with corresponding EEG activity in a male *Kcna1* ^–/–^ mouse. The start of the seizure is denoted with the red arrow. Incidence of pacing‐induced ventricular tachyarrhythmia (VTA) at baseline (*G*) and following isoproterenol (ISO) administration (*H*) in male (*n* = 9) and female (*n* = 20) WT mice and male (*n* = 7) and female (*n* = 13) *Kcna1* ^–/–^ mice. The number of VTA‐positive mice at baseline was 2/9 WT males, 5/7 KO males, 11/20 WT females and 6/13 KO females. After ISO, the number of VTA‐positive mice was 7/9 WT males, 4/7 KO males, 15/20 WT females and 4/13 KO females. Following ISO administration, female *Kcna1* ^–/–^ mice showed a significant decrease in VTA compared to their WT counterparts (*P* = 0.029). Brady, bradycardia; F, female; HR, heart rate; M, male; RMSSD, root mean square of successive beat‐to‐beat differences; SDNN, standard deviation of beat‐to‐beat intervals; Skips, cardiac conduction blocks/sinus pauses. *P < 0.05 (Fisher's exact test).

We next examined whether males have more severe autonomic or seizure‐related cardiac dysregulation that could be indicative of susceptibility to premature death by cardiac mechanisms. In an analysis of ECG recordings, *Kcna1^–/–^* mice revealed no significant sex differences in baseline HR during interictal periods (*P* = 0.841) (Fig. 2*A*). Similarly, we found no significant sex differences in interictal HRV when estimated using the time domain measures of SDNN (*P = *0.907), which reflects the index of total autonomic variability, and the root mean square of successive beat‐to‐beat differences (RMSSD) (*P = *0.756), which is an index of parasympathetic tone (Fig. 2*B* and *C*). We next analysed interictal HRV in the frequency domain, which also revealed a lack of sex differences in the 0.15–1.5 Hz (male *vs*. female: 3700 ± 362 ms^2^ Hz^−1^ *vs*. 2401 ± 1867 ms^2^ Hz^−1^; *P = *0.125, Wilcoxon signed‐rank) and 1.5–5 Hz (male *vs*. female: 2.3 ± 2.1 ms^2^ Hz^−1^ *vs*. 0.8 ± 0.8 ms^2^ Hz^−1^; *P = *0.126, Wilcoxon signed‐rank) frequency bands, which represent sympathetic and parasympathetic tone, respectively. Thus, autonomic control of the heart, as measured during interictal periods, is not a primary mechanism underlying the difference in survival rates between males and females.

We next quantified the incidence of seizure‐related cardiac abnormalities by analysing peri‐ictal ECG recordings. As in our previous studies (Dhaibar et al., 2019; Glasscock et al., 2010), we found that seizures in *Kcna1^–/–^* mice were often associated with cardiac conduction blocks or sinus pauses (i.e. skipped heartbeats) and bradycardia, but not tachycardia (Fig. 2*D*). In particular, females (*n* = 7; seizure count = 164) showed a significantly higher incidence of ictal bradycardia (23% *vs*. 13%; *P* = 0.041) (Fig. 2*D* and 2*E*), whereas males (*n* = 8; seizure count = 109) had slightly higher rates of preictal cardiac conduction blocks (4% *vs*. 0%; *P* = 0.025) (Fig. 2*D* and 2*F*). However, during the postictal period, when terminal cardiorespiratory failure typically initiates in patients, we found no significant sex differences in either the incidence of skipped heartbeats (*P* = 0.49) or bradycardia (*P* = 1).

### Female *Kcna1^–/–^* mice are resistant to inducible ventricular arrhythmias

In a previous study of the role of Kv1.1 in ventricular function, we found that adult *Kcna1^–/–^* mice exhibit resistance to intracardiac pacing‐induced VTA following administration of the β‐adrenergic agonist, isoproterenol, which normally enhances VTA susceptibility in WT mice (Trosclair et al., 2021). Because this prior analysis included both males and females grouped together, we retrospectively re‐analysed this dataset to test for sex differences. We found that male (*n* = 7) and female (*n* = 13) *Kcna1^–/–^* mice exhibited no significant differences in the incidence of VTA at baseline compared to their age‐matched WT counterparts (male *n* = 9, female *n* = 20) in response to intracardiac PES (Fig. 2*G*). However, following administration of isoproterenol, which should increase the likelihood of VTA, female *Kcna1^–/–^* mice exhibited significantly lower rates of VTA compared to their female WT counterparts (*P* = 0.029) (Fig. 2*H*). By contrast, male *Kcna1^–/–^* mice showed rates of VTA with isoproterenol that were not significantly different from WT males (*P* = 0.596). Thus, the overall reduction in VTA susceptibility that we observed previously in *Kcna1^–/–^* mice was mostly a result of females, which exhibited a sex‐specific resistance to VTA suggesting that they may have a lower risk for potentially harmful cardiac dysfunction related to sympathetic activation.

### Lethal seizures show similar patterns of cardiac dysfunction between sexes

During EEG‐ECG monitoring, we captured SUDEP events in a male and a female *Kcna1^–/–^* mouse (labelled male #5 and female #8) (Fig. 1*E*), providing insights into potential sex‐specific differences in the neurocardiac phenotypes of terminal seizures. The male *Kcna1^–/–^* mouse presented with seven seizures across 31 h of recording and had an average seizure duration of 43 s, which is slightly more than males as a group. The female *Kcna1^–/–^* mouse exhibited four seizures over a period of 3 h 15 min with an average seizure duration of 28 s, which was the lowest average seizure duration of any female recorded (Fig. 1*E*).

**Figure 3:**
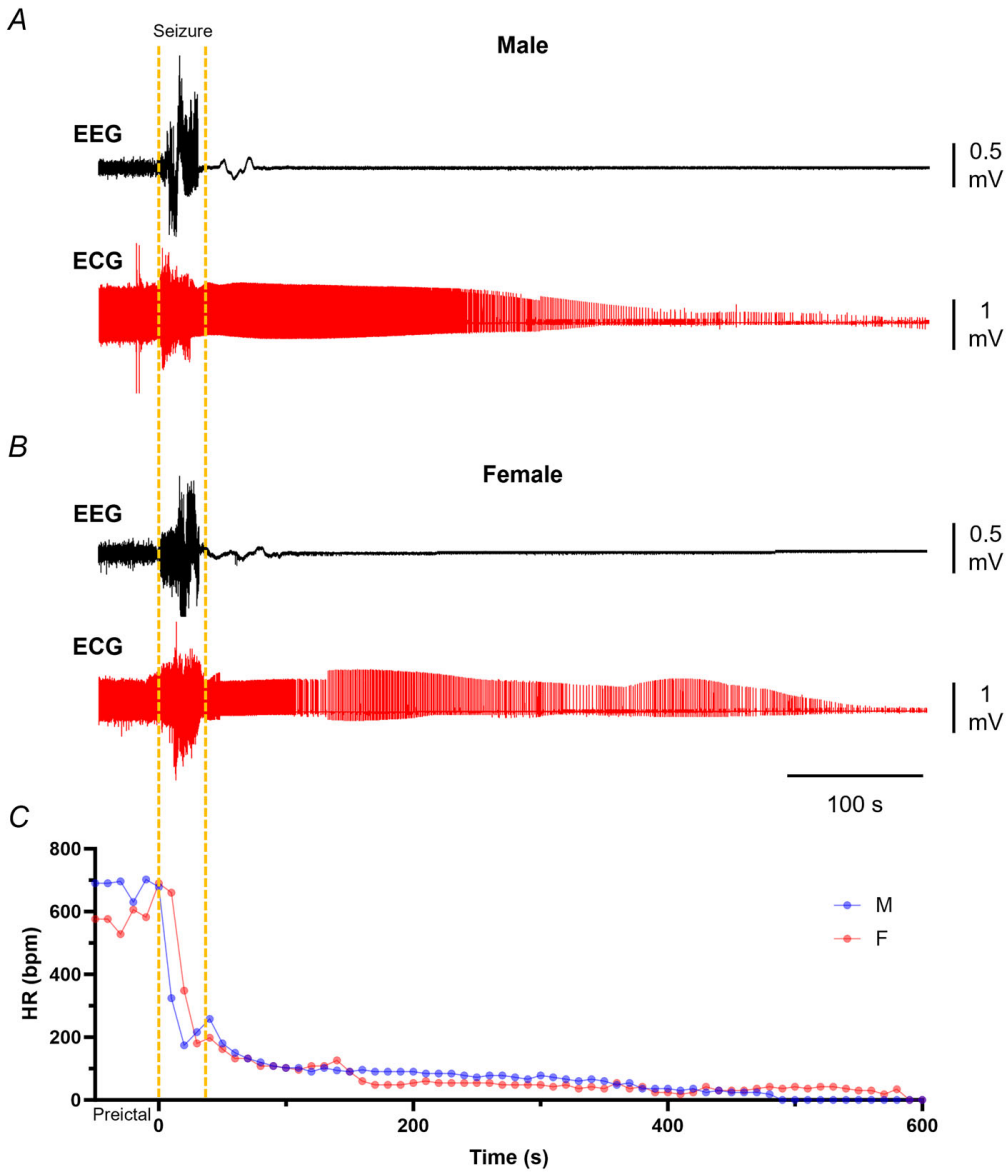
Male and female *Kcna1* ^–/–^ mice show similar neurocardiac phenotypes during lethal seizures Recording of simultaneous EEG‐ECG activity during a fatal spontaneous seizure in a male (*A*) and female (*B*) *Kcna1* ^–/–^ mouse. The top black trace indicates the neural EEG signal and the red trace represents the cardiac ECG signal. Onset and termination of the seizure are indicated by the dotted yellow lines. *C*, heart rates per minute (bpm) corresponding to the recording in (*A*) for the male and (*B*) for the female. Each dot in the plot represents a 10 s mean value. Time 0 represents seizure onset, and the pre‐ictal segment shows the 50‐s immediately preceding seizure onset. ECG, electrocardiography; EEG, electroencephalography; F, female; M, male.

The terminal seizures for both sexes lasted 39 s (denoted by the two yellow squares with orange outlines in Fig. 1*E*), ending with postictal generalized EEG suppression and a lack of EEG activity that never returned (Fig. 3*A* and *B*). Behaviourally, the two terminal seizures were extremely similar beginning with mild orofacial automatisms, abruptly transitioning to wild running and bouncing, and ending with tonic hindlimb extension and death (see Supporting information, Videos S1 and S2). The cardiac phenotypes were also largely similar between the sexes. Preictal cardiac activity was normal for both sexes, but, within 10–20 s of seizure onset, both the male and female showed 40–50% reductions in HR compared to the 50 s preictal values (Fig. 3*C*). The ictal bradycardia in both recordings continued to worsen postictally until ECG activity ceased altogether. Cessation of heart activity postictally occurred 490 s postictally for the male and 580 s postictally for the female (Fig. 3*C*).

Our recordings also captured one instance of lethal SE in a female *Kcna1^–/–^* mouse. This mouse exhibited 22 seizures during 16 h of recording with an average seizure duration of 87 s, not including the fatal SE event, well above the average seizure duration for the other *Kcna1^–/–^* female mice (Fig. 1*D*). The SE event started during the tenth recording hour and continued for 4 h 55 min. During this time, the EEG alternated between high‐frequency seizure‐like activity and individual sharp waves, with only brief intervals (<1 min) of relatively normal EEG patterns. Behaviourally, the mouse laid on its side with persistent leg twitching and never again resumed an upright position. From a cardiac perspective, the ECG showed progressively worsening bradycardia punctuated by skipped heartbeats. Eventually, the EEG permanently flatlined, but ECG activity continued for 910 s until cardiac failure.

### Seizures in female *Kcna1^–/–^* mice exhibit higher neurocardiac resetting strength

Given that traditional metrics of seizure severity, cardiac function and autonomic regulation revealed minimal sex differences, we hypothesized that more subtle distinctions in brain–heart communication between males and females might explain their disparate mortality rates. Studies on humans and rodents with epilepsy have demonstrated that seizures play a critical resetting function. In the lead‐up to a seizure, EEG dynamics show a progressive increase in spatiotemporal entrainment. Seizures reverse this synchronization, causing disentrainment of the EEG dynamics during the postictal period (Carney et al., 2004; Good et al., 2010; Hutson et al., 2018; Iasemidis et al., 2003, 2004; Sabesan et al., 2009). In more recent studies, we have identified reduced brain–heart association and connectivity in *Kcna1^–/–^* mice compared to WT mice, suggesting impaired neurocardiac coupling that increases the risk of SUDEP (Hutson et al., 2020; Mishra et al., 2017). Building on these previous discoveries, we tested a novel concept that seizures reset pathological neurocardiac connections more effectively in females than males, potentially mitigating dysregulation of the brain–heart axis that could increase the risk of seizure‐related death.

To investigate this hypothesis, we employed a network‐based organomics approach, treating the brain and heart as components of a single network and estimating their directional connectivity using GPDC to measure peri‐ictal changes in brain–heart communication. GPDC is a validated metric that provides an accurate measure of effective direct inflow between nodes (i.e. organs, such as brain → heart and heart → brain) in the frequency domain. We calculated the GPDC values for brain → heart and heart → brain interactions for each 10 s time segment throughout the EEG‐ECG recordings, as described previously (Hutson et al., 2020). To evaluate neurocardiac seizure resetting, we then compared preictal and postictal brain–heart GPDC connectivity during the 5 min intervals before and after seizures, respectively, by defining a SRI (see Methods). Positive SRI values indicate higher post‐ictal connectivity compared to pre‐ictal values, suggesting seizure resetting. Conversely, zero or negative SRI values indicate lower post‐ictal connectivity, implying an absence of seizure resetting.

**Figure 4:**
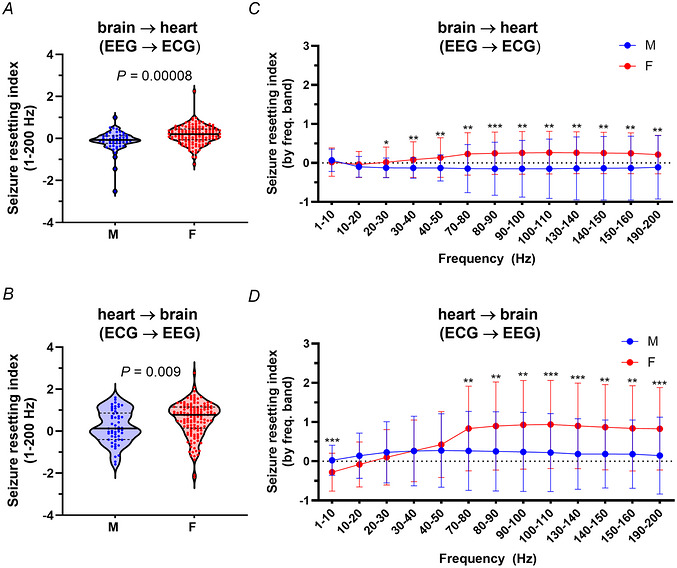
Seizures in female *Kcna1* ^–/–^ mice exhibit higher resetting of neurocardiac interactions Quantification of seizure resetting index (SRI) across the full frequency spectrum (1–200 Hz, excluding 60 Hz and its harmonic frequencies) shows that *Kcna1* ^–/–^ females have higher brain → heart (i.e. EEG → ECG) (*A*) and heart → brain (i.e. ECG → EEG) connectivity (*B*) in the post‐ictal period (5 min after seizure) relative to the pre‐ictal period (5 min before seizure), suggesting increased seizure resetting strength (*n* = 55 seizures in six males and *n* = 142 seizures in five females). The violin plots show the distribution of SRI data points with the median (continuous line) and interquartile values (dashed lines) indicated. Comparison of SRI values (mean ± SD) for each 10 Hz frequency band (excluding 60 Hz and its harmonics) for brain → heart (*C*) and heart → brain interactions (*D*) reveal frequency‐dependent effects with the enhanced resetting in females occurring most prominently at higher than 70 Hz in both pathways (*n* = 55 seizures averaged from six males and *n* = 142 seizures averaged in five females). The SD values of the SRI data points were calculated from the generated (resampled) seizure datasets per sex according to the bootstrap statistical methodology described in the Methods section. M, male; F, female. *P < 0.05; **P < 0.01; ***P < 0.001 (bootstrap resampling method).

This analysis of peri‐ictal brain–heart connectivity revealed significant sex differences in brain–heart communication patterns. When averaged across the full 200 Hz frequency spectrum (the 60 Hz band and its harmonic frequencies excluded), females exhibited significantly higher SRI values than males for both brain → heart and heart → brain interactions (*P = *0.00008 and *P* = 0.009, respectively) (Fig. 4*A* and *B*). Specifically, females showed positive SRI values for both directions of brain–heart interactions, suggesting effective neurocardiac resetting. By contrast, males displayed lower and even negative SRI values, particularly for brain → heart interactions, suggesting a lack of successful resetting.

To further investigate the frequency dependence of the observed resetting, we analysed the average SRI values per frequency bin within each 10 Hz frequency band between 1–200 Hz, excluding 60 Hz and its harmonics (Fig. 4*C* and *D*). We found that the enhanced resetting in females was most pronounced at higher frequencies: above 20 Hz for brain → heart interactions and above 80 Hz for heart → brain interactions. Although females exhibited significantly higher SRI values than males across almost all frequency bands, there was one notable exception: below 40 Hz, females showed less resetting than males for heart → brain interactions. These findings suggest that seizure‐induced resetting of brain–heart communication is both sex‐specific and frequency‐dependent.

We next tested whether the frequency‐dependent sex differences in SRI could be used to reliably predict the sex of a mouse, which would support the potential of SRI as a biomarker for stratifying SUDEP risk. To do this, we performed LDA using SRI values as predictors and the mouse's sex as the response variable, and evaluated prediction accuracy with leave‐one‐out cross‐validation. To enhance predictiveness, we included in our LDA model two heart → brain frequency bands (1–10 Hz and 100–110 Hz) and one brain → heart frequency band (80–90 Hz), selected for their highly significant sex differences in mean SRI. The model performed well, accurately predicting the sex of nine out of 11 mice in the study (82%), with 80% sensitivity (4/5) and 83% specificity (5/6) for females and 83% sensitivity (5/6) and 80% specificity (4/5) for males. Sensitivity represents the percentage of true positives, whereas specificity indicates the percentage of true negatives. These results demonstrate that SRI not only varies significantly between sexes, but also serves as a strong predictor of sex and, by extension, could be valuable for assessing SUDEP risk.

To explore the idea of neurocardiac resetting in more detail, we next examined the number of seizures that exhibit statistically significant resetting in each sex by measuring the PRS in each group. A seizure was considered to reset if its *T*‐index exceeded the threshold (*T*_th_) of 0.5273 (see Methods). We found that, like SRI values, females exhibited a larger PRS than males for frequencies above 20 Hz in the brain → heart direction (Table 2) and above 80 Hz in the heart → brain direction (Table 3). These results imply that, not only do seizures in females result in higher average postictal brain–heart connectivity (higher SRI) than males, but also the proportion of seizures that exhibit high levels of resetting is significantly greater in females than males.

**Table 2:** PRS in the brain → heart (EEG → ECG) pathway

Frequency (Hz)	Female (F) (PRS, SD)	Male (M) (PRS, SD)	F–M (PRS, SD)	*P* value of F–M
**1–10**	0.085, 0.023	0.073, 0.035	0.012, 0.043	0.807
**11–20**	0.056, 0.019	0.036, 0.026	0.020, 0.034	0.587
**21–30**	0.106, 0.026	0, 0	0.106, 0.026	0.013
**31–40**	0.127, 0.027	0, 0	0.127, 0.027	0.007
**41–50**	0.190, 0.033	0.018, 0.015	0.172, 0.038	0.004
**71–80**	0.303, 0.038	0.036, 0.021	0.266, 0.046	0.001
**81–90**	0.317, 0.039	0.073, 0.036	0.244, 0.054	0.002
**91–100**	0.324, 0.039	0.091, 0.040	0.233, 0.057	0.002
**101–110**	0.338, 0.039	0.091, 0.040	0.247, 0.057	0.001
**131–140**	0.324, 0.038	0.127, 0.047	0.197, 0.062	0.007
**141–150**	0.324, 0.039	0.127, 0.047	0.197, 0.062	0.009
**151–160**	0.317, 0.039	0.127, 0.047	0.190, 0.062	0.009
**191–200**	0.232, 0.036	0.109, 0.044	0.123, 0.058	0.059
**Full spectrum**	0.190, 0.031	0.018, 0.015	0.172, 0.037	0.004

*P* values were derived from the resulting empirical distribution for PRS.

Abbreviations: F, female; M, male; PRS, proportion of resetting seizures; SD, standard deviation of the proportions estimated per constructed (resampled) group of animals.

**Table 3:** PRS in the heart→brain (ECG→EEG) pathway

Frequency (Hz)	Female (F) (PRS, SD)	Male (M) (PRS, SD)	F–M (PRS, SD)	*P* value of F–M
**1–10**	0.028, 0.014	0.091, 0.040	−0.063, 0.043	0.067
**11–20**	0.106, 0.029	0.236, 0.055	−0.131, 0.063	0.029
**21–30**	0.225, 0.037	0.382, 0.066	−0.156, 0.076	0.033
**31–40**	0.331, 0.040	0.436, 0.066	−0.105, 0.078	0.174
**41–50**	0.472, 0.043	0.436, 0.066	0.035, 0.079	0.673
**71–80**	0.690, 0.041	0.382, 0.065	0.308, 0.077	0.001
**81–90**	0.669, 0.042	0.382, 0.065	0.287, 0.078	0.001
**91–100**	0.676, 0.042	0.382, 0.065	0.294, 0.078	0.002
**101–110**	0.669, 0.042	0.382, 0.065	0.287, 0.078	0.002
**131–140**	0.655, 0.042	0.345, 0.063	0.309, 0.077	0.001
**141–150**	0.627, 0.043	0.327, 0.063	0.299, 0.077	0.001
**151–160**	0.641, 0.042	0.309, 0.062	0.332, 0.076	0.001
**191–200**	0.641, 0.042	0.364, 0.064	0.277, 0.077	0.002
**Full spectrum**	0.585, 0.043	0.382, 0.065	0.203, 0.078	0.017

*P* values were derived from the resulting empirical distribution for PRS.

Abbreviations: F, female; M, male; PRS, proportion of resetting seizures; SD, standard deviation of the proportions estimated per constructed (resampled) group of animals.

Finally, we investigated the longer‐term temporal dynamics of neurocardiac seizure resetting to assess its stability and duration in relation to sex. Our analysis focused on peri‐ictal changes in brain–heart connectivity (GPDC values) within the 130–140 Hz frequency band, spanning 30 min before and after seizures. We selected this frequency band due to its pronounced and significant sex differences in SRI and PRS (Fig. 4 and Tables 2 and 3) in both brain → heart and heart → brain directions. To avoid confounding results, we only included seizures that occurred at least 1 h apart, ensuring non‐overlapping pre‐ and postictal windows. This exclusion criterion led to significantly less seizures analysed in males, which tended to have shorter inter‐seizure intervals as a result of seizures occurring in closer temporal proximity to one another (Fig. 1*G*).

**Figure 5:**
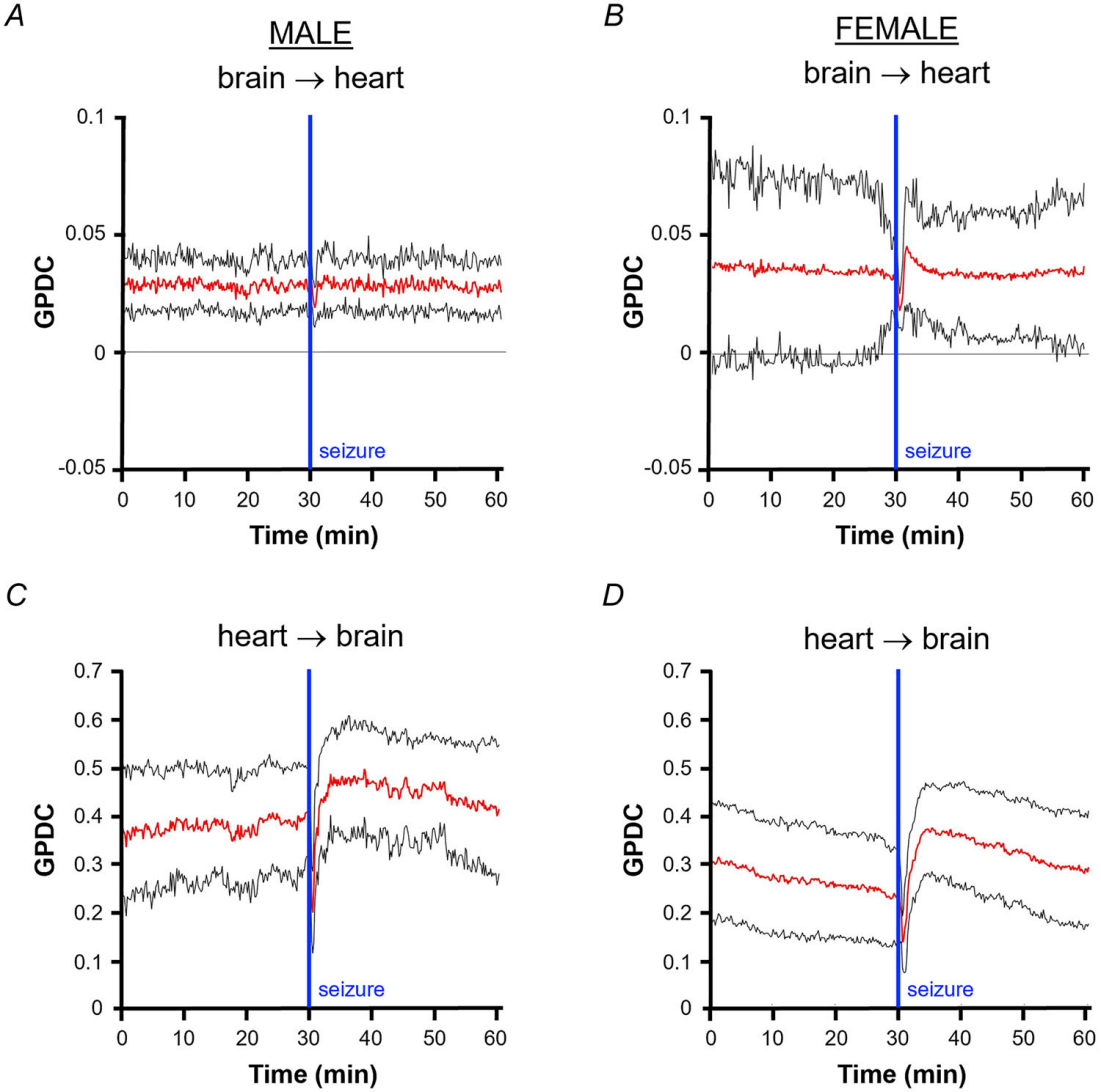
Analysis of the temporal dynamics of neurocardiac seizure resetting reveals sex‐specific differences in brain–heart connectivity Longer‐term peri‐ictal changes in neurocardiac interactions were assessed by comparing brain–heart generalized partial directed coherence (GPDC) values within the 130–140 Hz frequency band in male (*n* = 6; number of seizures = 55) and female (*n* = 5; number of seizures = 142) *Kcna1* ^–/–^ mice during the 30 min before (preictal) and 30 min after (postictal) seizures. Seizure onset is indicated by vertical blue lines; typically, seizures lasted no more than a minute (see Fig. 1*D*). The plots show the average GPDC value over time (red lines) ± SD (black lines). *A* and *B*, connectivity changes over time for the brain → heart pathway in *Kcna1* ^–/–^ males and females, respectively. Females exhibited slightly higher peri‐ictal brain → heart GPDC values than males, which increased further but transiently postictally. *C* and *D*, much higher connectivity values and resetting for the heart → brain pathway compared to the brain → heart pathway in both *Kcna1* ^–/–^ males and females. However, females exhibited a progressive drop in heart → brain GPDC values as the seizure was approaching.

Brain → heart connectivity analysis in the 130–140 Hz frequency band revealed that males had consistently lower GPDC values (and hence connectivity) than the females both pre‐ and postictally, with no evidence of seizure resetting (i.e. postictal values did not increase compared to preictal values) (Fig. 5*A* and *B*). By contrast, females exhibited slightly higher peri‐ictal brain → heart GPDC values, which further increased during the immediate postictal period; however, this increase was transient, returning to preictal levels within ∼5 min after seizures.

Heart → brain connectivity analysis in the 130–140 Hz frequency band showed that both sexes exhibited seizure resetting and that the peri‐ictal connectivity of this pathway was much higher than that of the brain → heart direction (Fig. 5*C* and *D*). However, females showed a progressive reduction in heart → brain GPDC values during the preictal period leading to seizure onset, a pattern absent in males.

## Discussion

The present study provides the first comprehensive assessment of sex differences in *Kcna1^–/–^* mice in the context of epilepsy and SUDEP. Most notably, we found that males had significantly higher mortality rates than females, but this was not simply a result of differences in epilepsy severity or seizure susceptibility. Instead, our research revealed sex‐specific cardiac responses to seizures, with females more probably experiencing bradycardia during spontaneous seizures. We also recorded SUDEP events in both a male and female mouse, which revealed similar patterns of profound bradyarrhythmic cardiac dysfunction beginning in the ictal period and leading to death. Retrospective analysis of intracardiac pacing data revealed that females exhibit resistance to VTA, further supporting sex differences in susceptibility to cardiac dysfunction. Using an organomics approach to model neurocardiac interactions, we discovered that seizures affect brain–heart communication differently between sexes. Our modelling suggests that seizures more effectively reset brain–heart interactions in females, providing a potential explanation and biomarker for their lower SUDEP risk. These findings highlight the complex interplay between sex, cardiac function and brain–heart communication in determining SUDEP susceptibility.

Our finding that female *Kcna1^–/–^* mice have lower SUDEP rates than males underscores the potential impact of genetics on sex‐specific SUDEP susceptibility. In a previous study of *Kcna1^–/–^* mice on a different genetic background (C3HeB/FeJ, *vs*. our Tac:N:NIHS‐BC), no sex‐based differences in lifespan were found (Chun et al., 2018). In that study, sex‐based survival differences only emerged when the mice were fed a ketogenic diet, which resulted in females showing prolonged survival relative to males (Chun et al., 2018). Furthermore, in our study, we observed SUDEP events beginning in the third week of life, whereas C3HeB/FeJ *Kcna1^–/–^* mice start dying later, between 4 and 5 weeks of age (Chun et al., 2018; Simeone et al., 2016). Additionally, ∼25% of our Tac:N:NIHS‐BC *Kcna1^–/–^* mice ultimately survive, whereas 100% of C3HeB/FeJ *Kcna1^–/–^* mice succumb to SUDEP by around 60 days old (Chun et al., 2018; Simeone et al., 2016). These differences in SUDEP rates and timelines between *Kcna1^–/–^* mice carrying the same mutation on different genetic backgrounds demonstrate the powerful influence of both sex and genetics on SUDEP risk.

Studies of other SUDEP mouse models further emphasize the role of genetics in determining sex‐specific SUDEP susceptibility. In *Scn8a^N1768D/+^* mice, females show longer lifespans (129 days) than males (96 days) despite similar seizure frequencies (Bahramnejad et al., 2024). Cannabinoid treatment of *Scn8a^N1768D/+^* mice has sex‐specific effects, shortening survival in females but prolonging it in males (Bahramnejad et al., 2024). In *Scn1a^+/−^* mice, unlike the *Kcna1* and *Scn8a* models, females show higher SUDEP rates than males (50% mortality at ∼24 days in females *vs*. ∼54 days in males), despite having slightly lower seizure frequencies (Niibori et al., 2020). Gene therapy improves survival in females without affecting seizure frequency, whereas, in males, it reduces seizure frequency without improving survival (Niibori et al., 2020). In DBA/1 mice, no sex differences are observed in susceptibility to seizure‐induced respiratory arrest and death in response to acoustic stimulation (Faingold & Randall, 2013). In mice with the K^+^/Cl^−^ co‐transporter KCC2 conditionally deleted in corticotropin‐releasing hormone neurons, SE‐induced chronic epilepsy results in high SUDEP rates in males but not females (Basu et al., 2024). These varied findings across different genetic models suggest that SUDEP susceptibility is not consistently higher in one sex. Instead, genetics play a crucial role in determining which sex is at higher risk, the timeframe of risk and sex‐specific responses to treatments.

Our observation of higher SUDEP incidence in male mice aligns with initial human studies that identified being male as a risk factor for SUDEP (Nilsson et al., 1999). However, more recent human studies and retrospective analysis of initial studies on sex and SUDEP have failed to replicate this association, casting doubt on whether a sex difference in SUDEP risk truly exists in humans (McHugh & Delanty, 2008; Tarighati Rasekhi et al., 2021). The variability in sex‐specific SUDEP risk observed across different genetic mouse models suggests a more nuanced relationship. It is possible that sex is not an independent risk factor for SUDEP, but rather interacts with genetic factors to influence risk in certain contexts (Hophing et al., 2022). This complexity may explain the inconsistent findings in human studies. Despite the current uncertainty in human data, the Centers for Disease Control still lists being male as a possible SUDEP risk factor (https://www.cdc.gov/epilepsy/sudep). Our results, combined with the mixed human data in the literature, underscore the need for further research that incorporates novel and more holistic analytical approaches (e.g. organomics) to clarify how sex and genetics influence SUDEP risk, potentially enabling more accurate risk assessments and targeted interventions.

Our findings obtained in the present study provide insights into how cardiac function is affected during fatal and non‐fatal seizures in male and female *Kcna1^–/–^* mice. We observed several sex‐specific differences in non‐fatal seizures. Male *Kcna1^–/–^* mice exhibited a greater proportion of preictal skipped heartbeats than females, although this occurred in only ∼5% of male seizures. By contrast, female *Kcna1^–/–^* mice experienced ictal bradycardia in ∼25% of their seizures, significantly more often than males. Additionally, in cardiac pacing experiments, female *Kcna1^–/–^* mice showed resistance to VTA compared to WT controls. This enhanced resistance to tachyarrhythmias may explain the prevalence of bradycardia, rather than tachycardia, during seizures in females. Despite sex‐specific differences in non‐fatal seizures, the two SUDEP events we captured showed similar cardiac phenotypes in both sexes. Terminal seizures lasted 39 s for both the male and female, ending in postictal EEG suppression with no resumption of brain activity. Profound bradycardia (40–50% reduction in HR from the preictal period) occurred within the first 10–20 s of the terminal seizure and continued postictally until heart activity ceased (490 s for males, 580 s for females). These SUDEP recordings are particularly valuable because only one other such event has been previously recorded with EEG‐ECG in a *Kcna1^–/–^* mouse. That study reported similar findings of consistent HR drops and periods of profound bradycardia during severe seizures, with worsening bradycardia following the terminal seizure until ECG activity ceased (Glasscock et al., 2010). Thus, our data suggest that, although males and females exhibit some mild peri‐ictal cardiac sex differences during non‐fatal seizures, the cardiac patterns in fatal seizures may be more similar between the sexes.

Organomics analysis revealed that neuro‐cardiac resetting occurs more effectively after seizures in females than in males, possibly contributing to the observed sex differences in seizure‐related mortality rates in *Kcna1^–/–^* mice. Previous research has demonstrated that seizures are preceded by increased entrainment and reduced chaoticity in brain networks that include the epileptogenic focus. The seizure event then resets chaoticity and disentrains the preictally entrained brain sites within the focus (Chakravarthy et al., 2009; Iasemidis, 2011), suggesting that seizures may act as a compensatory mechanism to correct the pathological brain behaviour that occurs in preictal periods. Given the complex interconnections between neural and cardiac control systems, we hypothesized that pathological preictal conditions probably affect not only communication between brain sites, but also the communication of the brain with the heart. Thus, we examined brain–heart connectivity as a measure of potentially abnormal preictal neurocardiac communication and its resetting postictally. Using peri‐ictal measures of seizure resetting (SRI and PRS), we found that, compared to males, female *Kcna1^–/–^* mice exhibited significantly larger postictal increases in brain–heart connectivity and a larger proportion of seizures that significantly reset the neurocardiac communications. Furthermore, predictive modelling demonstrated that SRI acts as a strong predictor of sex and, consequently, SUDEP susceptibility, highlighting its potential value as a biomarker for SUDEP risk stratification. Although we were not able to assess long‐term postictal stability of connections because of the limited period of observation and the high frequency of seizures, our results suggest that females demonstrate a greater probability than males of exhibiting increased brain–heart communication after seizures relative to their preictal state. This implies that some component of the neurocardiac control centre in females responds more significantly and positively to pathological preictal periods than in males. The sex differences in peri‐ictal cardiac dysfunction identified by manual examination of the EEG‐ECG (Fig. 2*D*) further suggest that the autonomic nervous system is differentially activated by seizures in males and females leading to sex‐specific cardiac effects. In light of the above findings, we propose a scenario where mortality risk in epilepsy is enhanced when a seizure fails to significantly repair or improve the reduced preictal brain–heart communication, potentially contributing to cardiorespiratory failure. Thus, the identified changes in peri‐ictal brain–heart connectivity could represent new biomarkers for SUDEP risk.

A limitation of the present study was its focus solely on the brain–heart axis, excluding potential sex differences in breathing. This focus was deliberate, driven by the need for reliable quantification of seizure phenotypes, which requires longer recording epochs to capture sufficient seizures for meaningful comparisons. While EEG‐ECG allows for continuous recordings exceeding 24 h, concurrent respiratory recordings are typically limited to 6‐ to 8‐h sessions due to plethysmography chamber constraints. Consequently, we were not able to draw conclusions about respiration's influence on the observed cardiac phenotypes or sex differences in SUDEP risk. This caveat is crucial, given that respiration has been proposed as a primary driver of SUDEP in both mice and humans (Dhaibar et al., 2019; Jansen et al., 2019; Kim et al., 2018; Ryvlin et al., 2013; Wenker et al., 2021). In *Kcna1* ^–/–^ mice, respiratory dysfunction may precede and potentially trigger cardiac dysfunction during seizures, potentially increasing SUDEP risk (Dhaibar et al., 2019). However, a cursory retrospective analysis of respiratory data from our previous study (Dhaibar et al., 2019) suggested similar incidences of ictal respiratory abnormalities between males (*n* = 3) and females (*n* = 4), indicating that seizure‐related breathing dysfunction may not significantly differ between sexes (data not shown). While we intend to further investigate sex differences in breathing in future studies, this initial investigation prioritized the brain–heart axis to establish a foundational understanding of sex‐specific cardiac responses to seizures.

In summary, our study reveals significant sex differences in mortality rates in *Kcna1^–/–^* mice, despite traditional analysis of EEG and ECG showing no drastic distinctions in brain or heart function between sexes. By applying a novel organomics/network approach to the EEG‐ECG recordings that allowed mathematical analysis of the interactions between the brain and heart biosignals, we uncovered that seizures differentially affect the communication between the brain and heart in males and females. Specifically, females exhibited a greater increase in brain–heart interactions following seizures, suggesting the existence of more effective homeostatic physiological resetting mechanisms in females that may protect against SUDEP. Although the exact physiological mechanisms underlying this effect remain to be determined, the differences in pre‐ and postictal brain–heart interactions between high‐risk (males) and low‐risk (females) mice could serve as a new and potentially valuable biomarker of SUDEP susceptibility. Furthermore, our findings reinforce the importance of including both sexes in preclinical research (Clayton & Collins, 2014) and provide insight into the mechanisms underlying sex‐specific differences in SUDEP risk. Thus, these results help pave the way for more precise risk stratification strategies and sex‐specific interventions in epilepsy management.

## Additional information

## Competing interests

The authors declare that they have no competing interests.

## Author contributions

K.P. analysed data, generated figures and wrote and edited the manuscript. P.K. designed research studies and analysed data. The order of co‐first authors K.P. and P.K. was assigned based on contributions to writing. K.K. analysed data and generated figures. T.N.H. analysed data and edited the manuscript. N.M.G.‐H. conducted experiments. D.‐S.S., K.T., H.A.D. and P.D. analysed data. M.W. conducted experiments and analysed data. L.I. designed research studies, analysed data and wrote and edited the manuscript. E.G. conceived and designed the research studies and wrote the manuscript.

## Funding

This work was supported by the National Institutes of Health (R01NS100954 to EG; R01NS099188 to EG; and R01NS129643 to EG and LI).

## Supporting information




Peer Review History

Video S1

Video S2

## Data Availability

The data that support the findings of this study are available from the corresponding author upon reasonable request.
