# Supplementary material for: Sex‐specific differences in mortality and neurocardiac interactions in the Kv1.1 knockout mouse model of sudden unexpected death in epilepsy (SUDEP)
Source: J Physiol. 2025 Jan 8;604(16):6731–51. doi: 10.1113/JP287582 (PMC13481012; doi:10.1113/JP287582)
Supplement: Supplementary file 1 [file TJP-604-6731-s001.pdf]

The Journal of Physiology

Sex-specific differences in mortality and neurocardiac interactions in the Kv1.1 knockout mouse model of sudden unexpected death in epilepsy (SUDEP)

Kelsey Paulhus, Praveen Kumar, Kelly Kneale, Noah Hutson, Nicole M. Gautier-Hall, Deng-Shan Shiau, Megan Watts, Krystle Trosclair, Hemangini A Dhaibar, Paari Dominic, Leonidas Iasemidis, and Edward Glasscock
DOI: 10.1113/JP287582

Corresponding author(s): Edward Glasscock (eglasscock@smu.edu)

The referees have opted to remain anonymous.

Review Timeline:

Submission Date:	29-Aug-2024
Editorial Decision:	03-Oct-2024
Revision Received:	21-Nov-2024
Editorial Decision:	03-Dec-2024
Revision Received:	11-Dec-2024
Accepted:	12-Dec-2024

Senior Editor: Bjorn Knollmann

Reviewing Editor: Daniel Zoccal

Transaction Report:

(Note: With the exception of the correction of typographical or spelling errors that could be a source of ambiguity, letters and reports are not edited. Depending on transfer agreements, referee reports obtained elsewhere may or may not be included in this compilation. Referee reports are anonymous unless the Referee chooses to sign their reports.)

Dear Dr Glasscock,

Re: JP-RP-2024-287582 "Sex-specific differences in mortality and neurocardiac interactions in the Kv1.1 knockout mouse model of sudden unexpected death in epilepsy (SUDEP)" by Kelsey Paulhus, Praveen Kumar, Kelly Kneale, Noah Hutson, Nicole M. Gautier-Hall, Deng-Shan Shiau, Megan Watts, Krystle Trosclair, Hemangini A Dhaibar, Paari Dominic, Leonidas Iasemidis, and Edward Glasscock

Thank you for submitting your manuscript to The Journal of Physiology. It has been assessed by a Reviewing Editor and by 2 expert referees and we are pleased to tell you that it is potentially acceptable for publication following satisfactory major revision.

Please advise your co-authors of this decision as soon as possible.

The referee reports are copied at the end of this email.

Please address all the points raised and incorporate all requested revisions or explain in your Response to Referees why a change has not been made. We hope you will find the comments helpful and that you will be able to return your revised manuscript within 2 months. If you require longer than this, please contact journal staff: jp@physoc.org. Please note that this letter does not constitute a guarantee for acceptance of your revised manuscript.

Your revised manuscript should be submitted online using the link in your Author Tasks: Link Not Available. This link is accessible via your account as Corresponding Author; it is not available to your co-authors. If this presents a problem, please contact journal staff (jp@physoc.org). Image files from the previous version are retained on the system. Please ensure you replace or remove any files that are being revised.

If you do not wish to submit a revised version of your manuscript, you must inform our journal staff (jp@physoc.org) or reply to this email to request withdrawal. Please note that a manuscript must be formally withdrawn from the peer review process at one journal before it may be submitted to another journal.

TRANSPARENT PEER REVIEW POLICY: To improve the transparency of its peer review process, The Journal of Physiology publishes online as supporting information the peer review history of all articles accepted for publication. Readers will have access to decision letters, including Editors' comments and referee reports, for each version of the manuscript, as well as any author responses to peer review comments. Referees can decide whether or not they wish to be named on the peer review history document.

ABSTRACT FIGURES: Authors are expected to use The Journal's premium BioRender account to create/redraw their Abstract Figures. Information on how to access this account is here: https://physoc.onlinelibrary.wiley.com/journal/14697793/biorender-access.

This will enable Authors to create and download high-resolution figures. If authors have used the free BioRender service, they can use the instructions provided in the link above to download a high-resolution version suitable for publication.

The link provided should only be used for the purposes of this submission. Authors will be charged for figures created on this account if they are not related to this manuscript submission.

LANGUAGE EDITING AND SUPPORT FOR PUBLICATION: If you would like help with English language editing, or other article preparation support, Wiley Editing Services offers expert help, including English Language Editing, as well as translation, manuscript formatting, and figure formatting at www.wileyauthors.com/eeo/preparation. You can also find resources for Preparing Your Article for general guidance about writing and preparing your manuscript at www.wileyauthors.com/eeo/prepresources.

RIGOUR AND REPRODUCIBILITY: We recommend authors consult The Journal's Rigour and Reproducibility requirements to ensure that their manuscript is suitable for publication: https://physoc.onlinelibrary.wiley.com/pb-assets/hub-assets/physoc/documents/TJP-Rigour-and-Reproducibility-Requirements.pdf

REVISION CHECKLIST:

Check that your Methods section conforms to journal policy: https://jp.msubmit.net/cgi-bin/main.plex?form_type=display_requirements#methods.

Check that data presented conforms to the statistics policy: https://jp.msubmit.net/cgi-bin/main.plex?form_type=display_requirements#statistics.

Upload a full Response to Referees file. To create your 'Response to Referees': copy all the reports, including any comments from the Senior and Reviewing Editors, into a Microsoft Word, or similar, file and respond to each point, using font or background colour to distinguish comments and responses and upload as the required file type.

Please upload two versions of your manuscript text: one with all relevant changes highlighted and one clean version with no changes tracked. The manuscript file should include all tables and figure legends, but each figure/graph should be uploaded as separate, high-resolution files.

You may also upload:

- 'Potential Cover Art' for consideration as the issue's cover image
- Appropriate Supporting Information (Video, audio or data set: see https://jp.msubmit.net/cgi-bin/main.plex?form_type=display_requirements#supp).

We look forward to receiving your revised submission.

If you have any queries, please reply to this email and we will be pleased to advise.

Yours sincerely,

Bjorn Knollmann
Senior Editor
The Journal of Physiology

REQUIRED ITEMS

- Author photo and profile. First or joint first authors are asked to provide a short biography (no more than 100 words for one author or 150 words in total for joint first authors) and a portrait photograph. These should be uploaded and clearly labelled together in a Word document with the revised version of the manuscript. See Information for Authors for further details.

- You must start the Methods section with a paragraph headed Ethical approval (https://jp.msubmit.net/cgi-bin/main.plex?form_type=display_requirements#methods).

Research must comply with The Journal's policies regarding animal experiments (https://physoc.onlinelibrary.wiley.com/hub/animal-experiments) and adherence to these policies must be stated in the manuscript.

Authors should confirm in their Methods section that their experiments were carried out according to the guidelines laid down by their institution's animal welfare committee, including an ethics approval reference number. The Methods section must contain a statement about access to food, water and housing, details of the anaesthetic regime: anaesthetic used, dose and route of administration, and method of killing the experimental animals.

- The reference list must be in alphabetical order, rather than numbered, to comply with our Journal format.

- Your manuscript must include a complete Additional Information section, including competing interests; funding; author contributions and acknowledgements.

- Please upload separate high-quality figure files via the submission form.

- Please ensure that any tables are editable and in Word format, and wherever possible, embedded in the article file itself.

- Please ensure that the Article File you upload is a Word file.

- Your paper contains Supporting Information of a type that we no longer publish, including supplementary tables and

figures. Any information essential to an understanding of the paper must be included as part of the main manuscript and figures. The only Supporting Information that we publish are video and audio, 3D structures, program codes and large data files. Your revised paper will be returned to you if it does not adhere to our Supporting Information Guidelines.

- Papers must comply with the Statistics Policy: https://jp.msubmit.net/cgi-bin/main.plex?form_type=display_requirements#statistics.

In summary:

- If n < 30, all data points must be plotted in the figure in a way that reveals their range and distribution. A bar graph with data points overlaid, a box and whisker plot or a violin plot (preferably with data points included) are acceptable formats.

- If n > 30, then the entire raw dataset must be made available either as supporting information, or hosted on a not-for-profit repository, e.g. FigShare, with access details provided in the manuscript.

- 'n' clearly defined (e.g. x cells from y slices in z animals) in the Methods. Authors should be mindful of pseudoreplication.

- All relevant 'n' values must be clearly stated in the main text, figures and tables.

- The most appropriate summary statistic (e.g. mean or median and standard deviation) must be used. Standard Error of the Mean (SEM) alone is not permitted.

- Exact p values must be stated. Authors must not use 'greater than' or 'less than'. Exact p values must be stated to three significant figures even when 'no statistical significance' is claimed.

- Please include an Abstract Figure file, as well as the Figure Legend text within the main article file. The Abstract Figure is a piece of artwork designed to give readers an immediate understanding of the research and should summarise the main conclusions. If possible, the image should be easily 'readable' from left to right or top to bottom. It should show the physiological relevance of the manuscript so readers can assess the importance and content of its findings. Abstract Figures should not merely recapitulate other figures in the manuscript. Please try to keep the diagram as simple as possible and without superfluous information that may distract from the main conclusion(s). Abstract Figures must be provided by authors no later than the revised manuscript stage and should be uploaded as a separate file during online submission labelled as File Type 'Abstract Figure'. Please also ensure that you include the figure legend in the main article file. All Abstract Figures should be created using BioRender. Authors should use The Journal's premium BioRender account to export high-resolution images. Details on how to use and access the premium account are included as part of this email.

EDITOR COMMENTS

Reviewing Editor:

Comments for Authors to ensure the paper complies with the Statistics Policy (Required):
Data were reported as mean{plus minus}SEM. To comply with our policy you must use SD rather than SEM.

Comments to the Author (Required):
I thank the authors for submitting their work to JPhysiol. Two experts in the field have assessed the manuscript. Both presented positive comments about the study; however, they also expressed concerns requiring the authors' attention, including the lack of animal model validation and clearer discussion about the functional relevance of the modeling analyses. These referees' comments sound positive and can improve the quality and relevance of the study. Besides, the authors must clearly describe how the anesthesia depth was monitored during the surgeries and the post-operative analgesia procedure employed in the manuscript. In data analyses, indicate if the data normal distribution was assessed before statistical analyses.

Please also see 'Required Items' above.

Senior Editor:

Comments for Authors to ensure the paper complies with the Statistics Policy (Required):
Please ensure the revision complies with the Statistics Policy

Comments to the Author:
Both the reviewer and the reviewing editor found merit in the study. I concur. However, as pointed out by the reviewing editor, a responsive revision will require experimental data to support the modeling results.

REFEREE COMMENTS

Referee #1:

This manuscript describes the results of investigating potential causes of premature deaths in a mouse model of epilepsy, the Kv1.1 knockout mouse. Importantly, the major focus of the investigation is on differences in male and female KO mice, which has historically been understudied and important for a more complete understanding of SUDEP in humans. The authors take advantage of previously collected data (mortality logs and intracardiac pacing data) and new in vivo and modeling experiments to explore sex differences in several measurements including the understudied concepts of the strength of connections between the brain and the heart and vice versa. This manuscript is very well written and clear and would be a significant addition to the preclinical literature regarding mechanisms of SUDEP. My comments/questions below are meant to enhance the final product and provide additional clarity.

Comments/questions:

1) Have the specific Kv1.1 KO mice used in this study been validated as knockouts via western blot or proteomics assays? If so, it would add strength to the rigor of the model and the comparisons drawn between this model and other Kv1.1 KO mice that have a different genetic background (apples to apples comparisons)?

2) While the focus is appropriately on mortality rates among male and female Kv1.1 KO mice in Figure 1, it would be worth mentioning what mortality rates were in male and female Hets and WT mice for this model. It may have already been published but I do not see a reference if that is the case. If not, perhaps these rates can be included in the Results section?

3) A minor point is equating the death events in this preclinical model to human SUDEP. As an example the authors in the final paragraph of the introduction (and perhaps in other sections) use the term "SUDEP rates" when referring to mortality in this model. I would caution the authors on equating the death events in this model to human SUDEP, and suggest softening the language to "SUDEP-like" events. Equating these deaths in mice to human SUDEP events feels like it would require significant validation that all aspects of human SUDEP are equal to this model. I say this as a researcher studying a different model of SUDEP. Alternatively, the term could be used if it is explicitly stated that "while some aspects of these death events are reminiscent those known in human SUDEP, we will use this term to describe death events in this model recognizing potential distinguishing characteristics may not reflect that which occurs in humans" or something like that.

4) In the introduction (paragraph 2) the third sentence is worded in a way that implies that it is well known that SUDEP occurs "...immediately following a seizure." This may seem like a minor point, but the human data on how long after a seizure death occurs is highly variable and largely unknown. Even in the MORTEMUS study the best estimates of the time between seizure termination and death (time to CPR mostly) is a few minutes to 3 hours. I suggest rewording the sentence such that it is more clear.

5) For the data captured form the 2 "SUDEP" events in your mice (Figure 3) I am wondering when would you say these animals died? I think you state the times for each event in which electrical activity of the heart ceased, but can you definitively state when (how long after the seizure) they died? Is this typical in this model? With only 2 observations it would be important to determine if these are "typical" or representative events.

6) I commend the authors for clearly describing the current state of the data regarding sex differences in human and preclinical models of SUDEP, and the lack of clarity. I think this is an important contribution to the literature and underappreciated!

Referee #2:

This study investigated possible sex-specific mechanisms associated with higher SUDEP susceptibility in males than females. To reach this goal, Paulhus et al. used Kcna1 knockout mice (Tac:N:NIHS-BC genetic background) as an experimental model for SUDEP, which shares similarities with humans. The authors used previous data to assess mortality in male and female KO animals and performed EEG and ECG analyses to identify potential sex-specific differences. According to the authors, the traditional seizure and cardiac analyses did not show relevant differences that could account for the higher SUDEP rate in males. However, the organomic analysis suggested that females exhibit a more significant brain-heart interaction resetting during the post-ictal period, which could be a protective mechanism against cardiorespiratory failure. The study is certainly relevant as it explores an important topic of investigation that is still not well understood. The methods employed were adequate, and the results provided valuable information. However, I couldn't visualize the functional relevance of the mathematical analyses of brain-heart signals in giving insights into the mechanisms for reducing SUDEP in females. Below are my comments for the authors' consideration.

1. The traditional EEG and ECG analyses exploring various seizure and cardiac metrics were clear and informative. In contrast, the organomic/network modeling data were not straightforward as several aspects of the analyses lack additional explanation regarding their significance and functional correlation. For example, I understood how the seizure resetting index was calculated; however, its physiological meaning is still unclear. Moreover, the functional importance of aggregating the frequency data in bands ranging from 1 to 200 Hz is not indicated (e.g., what is the relevance of the 130-140 Hz frequency band?).

2. The coherence analyses used to estimate brain-heart connectivity correlated EEG->ECG or ECG->EEG signals. In my understanding, this analysis demonstrated an association of events and not causality. Therefore, considering the complexity of multiple neural and hormonal mechanisms influencing the brain and heart interactions, the authors' view about this topic sounded superficial and lacked functional observations to support it. As a result, terms like "impaired neurocardiac coupling" or "effective reset brain-heart interaction" are not easily understandable. Besides, statements such as "some component of the neurocardiac control center in females responds more effectively and positively to pathological preictal periods than in males" sound vague. If the analyses suggest a protective cardiac effect in females, why did males and females show no relevant differences in cardiac function? Were the model analyses predictive, or did they reproduce a scenario of minimal differences between males and females? What do the authors mean by "more effective homeostatic physiological resetting mechanisms in females that may protect against SUDEP"? All these observations point to the need for proof-of-concept experiments to support the authors' conclusion. Otherwise, it sounds speculative.

3. If male and female KO mice showed similar cardiac dynamics, how is it possible to be sure that cardiac failure is the main cause of sex-related differences leading to SUDEP? As an additional analysis for HRV, the authors could have applied power spectral analyses to infer from the sympathetic and parasympathetic control of the heart in males and females. Besides, the authors briefly commented on preliminary data about the respiratory data in the discussion section, but it sounded relevant in the context of this study.

4. The authors mentioned that this study re-examined previous data from the research group. I identified citations to some studies in the manuscript, but it was not entirely clear if all the data used had already been published. For clarity, it would be essential if the authors indicate the source of these previous data in the Methods section.

END OF COMMENTS

RESPONSE TO REVIEWER COMMENTS

We thank the reviewers for their thoughtful and detailed evaluation of our manuscript. Below, we provide a point-by-point response to each comment in ***blue****.*

In response to the reviewers' feedback, we have made substantial modifications and enhancements throughout the manuscript, including key new experimental data. These updates include: ***(1) extensive new text*** *(highlighted in yellow in both this response and the manuscript) in the Abstract, Introduction, Methods, Results, and Discussion sections to address key points raised;* ***(2) new predictive modeling analyses*** *to evaluate the ability of seizure resetting measures to predict sex and, by extension, SUDEP risk; and* ***(3) new analyses examining heart rate variability*** *in the frequency domain.*

We believe the inclusion of these predictive modeling data is particularly impactful, as it provides robust support for the potential utility of seizure resetting measures as a biomarker for SUDEP risk stratification. By addressing the reviewers' comments in detail and incorporating these important changes, we have significantly strengthened the manuscript and are hopeful that it is now suitable for publication.

We sincerely appreciate the reviewers' insights, which have greatly improved the clarity and scientific rigor of this work.

EDITOR COMMENTS

Reviewing Editor:

Comments for Authors to ensure the paper complies with the Statistics Policy (Required):
Data were reported as mean{plus minus}SEM. To comply with our policy you must use SD rather than SEM.

We have changed all statistics in the manuscript to standard deviation to align with journal policy.

Comments to the Author (Required):
I thank the authors for submitting their work to JPhysiol. Two experts in the field have assessed the manuscript. Both presented positive comments about the study; however, they also expressed concerns requiring the authors' attention, including the lack of

animal model validation and clearer discussion about the functional relevance of the modeling analyses. These referees' comments sound positive and can improve the quality and relevance of the study. Besides, the authors must clearly describe how the anesthesia depth was monitored during the surgeries and the post-operative analgesia procedure employed in the manuscript. In data analyses, indicate if the data normal distribution was assessed before statistical analyses.

We appreciate the editors and reviewers for their thoughtful comments on our manuscript. We believe we have incorporated these comments and present a stronger manuscript in this resubmission. Please note we have now included the requested comments about anesthesia depth monitoring and post-operative analgesia procedures in our methods section. Furthermore, we have added in a line in the statistical methods section regarding testing for normal distribution in the data before statistical analysis. We write: “Normality was assessed prior to statistical analysis.”

Please also see 'Required Items'.

We have completed all the “Required Items” listed in the original decision email.

Senior Editor:

Comments for Authors to ensure the paper complies with the Statistics Policy (Required):
Please ensure the revision complies with the Statistics Policy

We have changed all statistics in the manuscript to standard deviation to align with journal policy.

Comments to the Author:
Both the reviewer and the reviewing editor found merit in the study. I concur. However, as pointed out by the reviewing editor, a responsive revision will require experimental data to support the modeling results.

We appreciate the editor’s complementary comment on our study. In this resubmission, we have included new text and new experimental data which we feel significantly strengthens the manuscript. Each change is detailed below with its corresponding reviewer comment.

REFEREE COMMENTS

Referee #1:

This manuscript describes the results of investigating potential causes of premature deaths in a mouse model of epilepsy, the Kv1.1 knockout mouse. Importantly, the major focus of the investigation is on differences in male and female KO mice, which has historically been understudied and important for a more complete understanding of SUDEP in humans. The authors take advantage of previously collected data (mortality logs and intracardiac pacing data) and new in vivo and modeling experiments to explore sex differences in several measurements including the understudied concepts of the strength of connections between the brain and the heart and vice versa. This manuscript is very well written and clear and would be a significant addition to the preclinical literature regarding mechanisms of SUDEP. My comments/questions below are meant to enhance the final product and provide additional clarity.

Thank you for your complimentary assessment of our manuscript and study.

Comments/questions:

1) Have the specific Kv1.1 KO mice used in this study been validated as knockouts via western blot or proteomics assays? If so, it would add strength to the rigor of the model and the comparisons drawn between this model and other Kv1.1 KO mice that have a different genetic background (apples to apples comparisons)?

We did not use western blots or proteomics to validate that the specific KO mice used in this study were KOs. Instead, we verified them using PCR analysis of genomic DNA from tail clips, as outlined in the Methods section. Furthermore, the presence of seizures in these serves as validation, as WT and heterozygous mice do not exhibit spontaneous seizures, as previously reported by Smart et al (DOI: 10.1016/s0896-6273(00)81018-1), and consistent with our own prior unpublished observations. Generating and comparing proteomics data from our Kv1.1 KO strain with data from other genetic backgrounds could offer valuable insights into how genetic background influences phenotype in this model. However, those experiments are beyond the scope of this initial study.

2) While the focus is appropriately on mortality rates among male and female Kv1.1 KO mice in Figure 1, it would be worth mentioning what mortality rates were in male and female Hets and WT mice for this model. It may have already been published but I do not see a reference if that is the case. If not, perhaps these rates can be included in the Results section?

We agree that addressing the mortality in wild type and heterozygous mice is important and have added the following new text in the Results section 3.1: "Importantly, we have

not observed premature lethality in wildtype (*Kcna1*$^{+/+}$) and heterozygous (*Kcna1*$^{+/-}$) littermate mice."

3) A minor point is equating the death events in this preclinical model to human SUDEP. As an example the authors in the final paragraph of the introduction (and perhaps in other sections) use the term "SUDEP rates" when referring to mortality in this model. I would caution the authors on equating the death events in this model to human SUDEP, and suggest softening the language to "SUDEP-like" events. Equating these deaths in mice to human SUDEP events feels like it would require significant validation that all aspects of human SUDEP are equal to this model. I say this as a researcher studying a different model of SUDEP. Alternatively, the term could be used if it is explicitly stated that "while some aspects of these death events are reminiscent those known in human SUDEP, we will use this term to describe death events in this model recognizing potential distinguishing characteristics may not reflect that which occurs in humans" or something like that.

We agree that the complexity of human SUDEP may not be exactly represented in our mouse model and did not intend to cause confusion with our wording. As suggested, we have added the following new text at the end of Results section 3.1 to clarify this: "Of note, since many aspects of these sudden death events in *Kcna1*$^{-/-}$ mice resemble SUDEP in humans, we will refer to these premature deaths as "SUDEP" in this study. However, we acknowledge that the mice may not model the full complexity of human SUDEP and that some deaths may result from status epilepticus."

4) In the introduction (paragraph 2) the third sentence is worded in a way that implies that it is well known that SUDEP occurs "...immediately following a seizure." This may seem like a minor point, but the human data on how long after a seizure death occurs is highly variable and largely unknown. Even in the MORTEMUS study the best estimates of the time between seizure termination and death (time to CPR mostly) is a few minutes to 3 hours. I suggest rewording the sentence such that it is more clear.

We agree that the timing of death in human SUDEP can be variable. To clarify this, we have reworded the sentence. The new text now reads: "The exact underlying causes of SUDEP are still unknown, but it is generally believed to result from seizure-related cardiorespiratory failure, typically occurring in the minutes to hours following a seizure, that is, during the postictal period (Devinsky, 2011)."

5) For the data captured from the 2 "SUDEP" events in your mice (Figure 3) I am wondering when would you say these animals died? I think you state the times for each event in which electrical activity of the heart ceased, but can you definitively state when (how long after the seizure) they died? Is this typical in this model? With only 2 observations it would be important to determine if these are "typical" or representative events.

We appreciate this important question. The clinical definition of death in humans is "an individual who has sustained either (1) irreversible cessation of circulatory and

respiratory functions, or (2) irreversible cessation of all functions of the entire brain, including the brain stem...” (DOI: 10.1057/978-1-137-53828-4_2). For the mice in Figure 3, both appear to be “brain dead” at the onset of the post-ictal period, as indicated by the terminal EEG flattening, which reflects the suppression of brain activity that does not return. While this could potentially be considered death due to irreversible cessation of normal brain activity, the EEG flattening may not necessarily mean that all brain functions have completely ceased at that moment. Furthermore, from a cardiac standpoint, the heart continues to beat for several minutes after the seizure in both cases before the onset of terminal asystole, which could also be considered the time of death. Because either brain death or cardiac arrest could serve as physiological markers of death, we chose not to specify a time of death for these mice in the paper, as it is unclear which metric is the most reliable and when exactly those functions become irreversibly ceased.

The SUDEP events recorded in this paper are among the few ever captured with EEG-ECG recordings. To our knowledge, the only other such event was reported in 2010, when we recorded an abnormally long (277 s) terminal seizure that was also associated with bradyarrhythmias in the post-ictal period (DOI:10.1523/JNEUROSCI.5591-09.2010). Given the small number of SUDEP events captured with EEG-ECG in this model, we cannot definitively say whether our observations are representative of the majority of death events in these mice. In the fifth paragraph of the Discussion, we compare our two recordings reported here to the previous observation: “These SUDEP recordings are particularly valuable, as only one other such event has been previously recorded with EEG-ECG in a *Kcna1*$^{-/-}$ mouse. That study reported similar findings of consistent heart rate drops and periods of profound bradycardia during severe seizures, with worsening bradycardia following the terminal seizure until ECG activity ceased (30).”

6) I commend the authors for clearly describing the current state of the data regarding sex differences in human and preclinical models of SUDEP, and the lack of clarity. I think this is an important contribution to the literature and underappreciated!

We genuinely appreciate this comment!

Referee #2:

This study investigated possible sex-specific mechanisms associated with higher SUDEP susceptibility in males than females. To reach this goal, Paulhus et al. used Kcna1 knockout mice (Tac:N:NIHS-BC genetic background) as an experimental model for SUDEP, which shares similarities with humans. The authors used previous data to assess mortality in male and female KO animals and performed EEG and ECG analyses to identify potential sex-specific differences. According to the authors, the

traditional seizure and cardiac analyses did not show relevant differences that could account for the higher SUDEP rate in males. However, the organomic analysis suggested that females exhibit a more significant brain-heart interaction resetting during the post-ictal period, which could be a protective mechanism against cardiorespiratory failure. The study is certainly relevant as it explores an important topic of investigation that is still not well understood. The methods employed were adequate, and the results provided valuable information. However, I couldn't visualize the functional relevance of the mathematical analyses of brain-heart signals in giving insights into the mechanisms for reducing SUDEP in females. Below are my comments for the authors' consideration.

We appreciate your recognition of our study's value and welcome the discussion of our mathematical approach's functional relevance. While the physiological mechanisms underlying our organomics analysis are not yet fully understood, this approach shows significant promise for identifying reliable SUDEP risk biomarkers. These biomarkers can enhance risk stratification efforts even as research continues to reveal their functional and physiological correlates. With recent grant support, our lab is actively investigating the biological foundations of these connectivity patterns, which we plan to report in future publications as those studies are completed. Our long-term goal is to translate these mathematical findings into explicit biological mechanisms, advancing toward a comprehensive understanding of sex-specific SUDEP risk factors.

1. The traditional EEG and ECG analyses exploring various seizure and cardiac metrics were clear and informative. In contrast, the organomic/network modeling data were not straightforward as several aspects of the analyses lack additional explanation regarding their significance and functional correlation. For example, I understood how the seizure resetting index was calculated; however, its physiological meaning is still unclear. Moreover, the functional importance of aggregating the frequency data in bands ranging from 1 to 200 Hz is not indicated (e.g., what is the relevance of the 130-140 Hz frequency band?).

We appreciate this thoughtful comment and agree that understanding the physiological mechanisms behind the organomics modeling and seizure resetting is important. In this manuscript, our primary objective is to report the significant finding that female mice demonstrate greater seizure resetting than male mice, suggesting that their seizures may more effectively counterbalance pathological brain activity in the preictal phase. Although this resetting mechanism is identified mathematically, it provides an initial framework for understanding a complex, systems-level response that may help mitigate SUDEP risk in females. Here, our focus is on introducing this novel resetting phenomenon as a potential biomarker for SUDEP risk stratification, a contribution we believe holds significant value even as the mechanistic details are being explored. Furthermore, the potential value of resetting as a biomarker is underscored by new analyses we have added to this revised manuscript. Specifically, we applied Linear Discriminant Analysis and leave-one-out cross-validation, which demonstrate that the resetting phenomenon serves as a highly predictive indicator of sex, and by extension,

may help stratify SUDEP risk (see our discussion below in response to your comment #2).

We aggregated the frequency data in 10-Hz bands for analysis to help identify frequency bands where changes in connectivity may be significant. We focused on the 130-140 Hz frequency band for further analysis (e.g., in Figure 5) not due to any known functional significance, but because it exhibited substantial directional differences (brain→heart and heart→brain) in Figure 4C-D. Given the pronounced sex differences within this band, we felt it would provide a clear visual representation of these dynamical sex differences. We provide a rationale for this in the Results section (paragraph 6 of section 3.7), stating: "Our analysis focused on peri-ictal changes in brain-heart connectivity (GPDC values) within the 130-140 Hz frequency band, spanning 30-min before and after seizures. We selected this frequency band due to its pronounced and significant sex differences in SRI and PRS (Figure 4, Table 2, 3) in both brain→heart and heart→brain directions".

Finally, our decision to analyze data up to 200 Hz was based on both technical considerations and alignment with our previous studies. From a technical standpoint, we set a conservative 200-Hz upper limit based on the Nyquist sampling theorem (the minimum sampling rate should be at least twice the highest frequency component present in the analog signal) to avoid aliasing. Hence, a sampling rate of 1 KHz avoids aliasing after the analog signal is filtered by a low-pass 500Hz cut-off filter. In our recordings, the raw EEG was sampled at 1 kHz, and the raw ECG was sampled at 2 kHz and subsequently down-sampled to 1 kHz to match the EEG for analysis, allowing for GPDC computation up to a theoretical maximum of 500 Hz. However, common practice suggests analyzing up to one-third of the sampling frequency instead of the theoretical maximum (half), which would be 333 Hz for our data. To be conservative, we chose to limit our calculations to 200 Hz. This range also aligns with previous studies (PMID: 34223181), ensuring comparability. To clarify this point, we have added the following new text in Methods section 2.7: "The decision to analyze data between 1-200 Hz was based on the Nyquist theorem. With a sampling rate of 1 kHz, the theorem suggests an upper limit of one-half the sampling rate, or one-third in common practice, which corresponds to frequencies up to 333 Hz in our data without aliasing. To be conservative, an upper limit of 200 Hz was applied for our calculations."

2. The coherence analyses used to estimate brain-heart connectivity correlated EEG->ECG or ECG->EEG signals. In my understanding, this analysis demonstrated an association of events and not causality. Therefore, considering the complexity of multiple neural and hormonal mechanisms influencing the brain and heart interactions, the authors' view about this topic sounded superficial and lacked functional observations to support it. As a result, terms like "impaired neurocardiac coupling" or "effective reset brain-heart interaction" are not easily understandable. Besides, statements such as "some component of the neurocardiac control center in females responds more effectively and positively to pathological preictal periods than in males" sound vague. If

the analyses suggest a protective cardiac effect in females, why did males and females show no relevant differences in cardiac function?

Thank you for this thoughtful question. To clarify, Generalized Partial Directed Coherence (GPDC) is mathematically derived from Granger Causality, meaning it is designed to estimate causal relationships between signals (DOI: https://doi.org/10.1098/rsta.2012.0158). We intentionally used more tentative language when describing the resetting phenomenon because we have not yet identified the specific physiological mechanisms underlying these newly observed patterns. As the reviewer rightly notes, brain-heart connectivity is likely influenced by complex neural and hormonal mechanisms, adding layers of intricacy to our interpretation of these results. Determining the exact functional correlations with brain-heart connectivity is a central objective of ongoing research in our lab, supported by a recently awarded grant. Uncovering the physiological basis behind these connectivity measures is a significant and complex challenge that we anticipate will require a series of future studies.

Regarding the absence of observable sex differences in traditional cardiac function metrics, we see this as reinforcing the unique strength of organomics analysis. Unlike traditional ECG analysis, organomics can uncover subtle, latent differences in brain-heart dynamics that may not manifest in surface-level cardiac function measures. We believe this speaks to the potential of organomics to reveal critical insights that traditional analyses may overlook.

Were the model analyses predictive, or did they reproduce a scenario of minimal differences between males and females?

We appreciate this question that enabled us to conduct additional new analyses to assess the predictive power of our model. Specifically, we tested whether the Seizure Resetting Index (SRI) could reliably predict the sex of a mouse. To do this, we used Linear Discriminant Analysis (LDA), with SRI values as predictors and the mouse's sex as the response variable. We assessed prediction accuracy using leave-one-out cross-validation. We have added the following text in section 2.8 to describe this process.

To investigate whether SRI could be used to predict the sex of a mouse, Linear Discriminant Analysis (LDA) was performed using the statistical software R (https://cran.r-project.org/doc/manuals/r-release/R-intro.html), with SRI values as predictors and the mouse's sex as the response variable. The SRI values included were one of the low and one of the high frequency bands in the heart→brain interaction (1-10 Hz and 100-110 Hz, respectively) and one of the high frequency band in the brain→heart frequency band (80-90 Hz), chosen based on their highest significant sex differences (positive or negative) in mean SRI (see **Figure 4**). Leave-one-out cross

validation was then applied to assess the prediction accuracy. The accuracy of the model for each sex was quantified using the following formula:

$$\text{Accuracy} = (\text{Sensitivity}^F \times N^F + \text{Specificity}^F \times N^M) / (N^F + N^M)$$

$$\text{or} = (\text{Sensitivity}^M \times N^M + \text{Specificity}^M \times N^F) / (N^F + N^M)$$

where N = the sample sizes of females (F) or males (M); Sensitivity = % of true positive; and Specificity = % of true negative.

The model performed well, correctly predicting the sex of 9 out of 11 mice (accuracy of 81.8%), with 80% sensitivity (4/5) and 83.3% specificity (5/6) for females, and 83.3% sensitivity (5/6) and 80% specificity (4/5) for males. Here, sensitivity represents the percentage of true positives, and specificity represents the percentage of true negatives.

These results indicate that SRI not only differs significantly between sexes but also serves as a strong predictor of sex and, by extension, could be valuable for assessing SUDEP risk. We have included these new results in Results section 3.7 and detailed the methods in Methods section 2.8. We have also added new text regarding this in the Discussion.

What do the authors mean by "more effective homeostatic physiological resetting mechanisms in females that may protect against SUDEP"? All these observations point to the need for proof-of-concept experiments to support the authors' conclusion. Otherwise, it sounds speculative.

Thank you for highlighting this point. The sentence in question appears at the end of the Discussion, where we intended to make a speculative, forward-looking statement about potential scientific implications of our findings. We purposely used tentative language (e.g., 'suggesting' and 'may') to indicate that this interpretation is hypothetical and open to further exploration. While direct proof-of-concept experiments to test these hypotheses would be ideal, they are extremely challenging, and we are not aware of any feasible experimental approaches that could be incorporated within the scope of this study. For this reason, we placed this statement at the end of the Discussion to signal that these interpretations are speculative and to set the stage for future studies that will be needed to fully explore and clarify the underlying mechanisms.

To clarify that the underlying physiology behind our organomics modeling is not yet understood, we have added new text in the Discussion following the referenced sentence to acknowledge this important limitation: "…females exhibited a greater increase in brain-heart interactions following seizures, suggesting the existence of more effective homeostatic physiological resetting mechanisms in females that may protect

against SUDEP. *Although the exact physiological mechanisms underlying this effect remain to be determined*, ..."

3. If male and female KO mice showed similar cardiac dynamics, how is it possible to be sure that cardiac failure is the main cause of sex-related differences leading to SUDEP? As an additional analysis for HRV, the authors could have applied power spectral analyses to infer from the sympathetic and parasympathetic control of the heart in males and females. Besides, the authors briefly commented on preliminary data about the respiratory data in the discussion section, but it sounded relevant in the context of this study.

We appreciate this question and did not intend to say that cardiac failure is the main cause of sex-related differences in SUDEP. What we are able to say with our data is that females show resistance to inducible ventricular tachyarrhythmias and have more ictal bradycardia, but during fatal seizures both male and female mice show similar patterns of cardiac arrest. Additionally, we see that the communication between the brain and heart (in both directions) is reset to more normal levels after seizures in females compared to males. We acknowledge that there could be other mechanisms at play that contribute to the sex-related differences seen in this study.

We appreciate the suggestion to look at power spectrum in our HRV data. As suggested, we performed these new analyses to look for autonomic sex differences in the heart. However, we still did not find statistically significant differences. We have included new text in section 3.4 of the results to show this additional analysis: "We next analyzed interictal HRV in the frequency domain, which also revealed a lack of sex differences in the 0.15-1.5 Hz (male vs. female: 3700 $\pm$ 362 ms^2/Hz vs. 2401 $\pm$ 1867 ms^2/Hz; P = 0.125, Wilcoxon signed-rank) and 1.5-5 Hz (male vs. female: 2.3 $\pm$ 2.1 ms^2/Hz vs 0.8 $\pm$ 0.8 ms^2/Hz; P = 0.126, Wilcoxon signed-rank) frequency bands, which represent sympathetic and parasympathetic tone, respectively."

4. The authors mentioned that this study re-examined previous data from the research group. I identified citations to some studies in the manuscript, but it was not entirely clear if all the data used had already been published. For clarity, it would be essential if the authors indicate the source of these previous data in the Methods section.

We appreciate this comment and have better clarified the source data. Our mouse survival log was previously reported in another paper (DOI: 10.1016/j.nbd.2020.104759) but there it was not analyzed by sex. We have added new text in the Methods clarifying this: "The lifespans of all KO mice in our colony were logged as standard lab protocol and used to generate the male and female survival curves. The lifespan data reported here have been shown in a previous publication without being separated by sex (Trosclair *et al.*, 2020); however, in this study the data were reanalyzed and the difference between males and females was measured and reported."

We also retrospectively analyzed pacing data and have already included the citation in the methods: "*In vivo* pacing studies of 4-month-old KO and WT mice (34) were retrospectively analyzed for sex differences." However, we did add the citation for this work into the Introduction where we reference it. We also reference a study in the discussion where we retrospectively analyzed respiratory data in the KO mice, but we have also included the reference in the sentence discussing the findings: "However, a cursory retrospective analysis of respiratory data from our previous study (29) suggested similar incidences of ictal respiratory abnormalities between males (n = 3) and females (n = 4), indicating that seizure-related breathing dysfunction may not significantly differ between sexes (data not shown)." All other data discussed in this study was uniquely generated for this specific project and has not been published before.

Dear Dr Glasscock,

Re: JP-RP-2024-287582R1 "Sex-specific differences in mortality and neurocardiac interactions in the Kv1.1 knockout mouse model of sudden unexpected death in epilepsy (SUDEP)" by Kelsey Paulhus, Praveen Kumar, Kelly Kneale, Noah Hutson, Nicole M. Gautier-Hall, Deng-Shan Shiau, Megan Watts, Krystle Trosclair, Hemangini A Dhaibar, Paari Dominic, Leonidas Iasemidis, and Edward Glasscock

Thank you for submitting your manuscript to The Journal of Physiology. It has been assessed by a Reviewing Editor and by 2 expert referees and we are pleased to tell you that it is acceptable for publication following satisfactory revision.

The referee reports are copied at the end of this email.

Please address all the points raised and incorporate all requested revisions or explain in your Response to Referees why a change has not been made. We hope you will find the comments helpful and that you will be able to return your revised manuscript within 4 weeks. If you require longer than this, please contact journal staff: jp@physoc.org.

Your revised manuscript should be submitted online using the link in your Author Tasks Link Not Available. This link is accessible via your account as Corresponding Author; it is not available to your co-authors. If this presents a problem, please contact journal staff (jp@physoc.org). Image files from the previous version are retained on the system. Please ensure you replace or remove any files that are being revised.

If you do not wish to submit a revised version of your manuscript, you must inform our journal staff (jp@physoc.org) or reply to this email to request withdrawal. Please note that a manuscript must be formally withdrawn from the peer review process at one journal before it may be submitted to another journal.

TRANSPARENT PEER REVIEW POLICY: To improve the transparency of its peer review process The Journal of Physiology publishes online, as supporting information, the peer review history of all articles accepted for publication. Readers will have access to decision letters, including Editors' comments and referee reports, for each version of the manuscript, as well as any author responses to peer review comments. Referees can decide whether or not they wish to be named on the peer review history document.

ABSTRACT FIGURES: Authors are expected to use The Journal's premium BioRender account to create/redraw their Abstract Figures. Information on how to access this account is here: https://physoc.onlinelibrary.wiley.com/journal/14697793/biorender-access.

This will enable Authors to create and download high-resolution figures. If authors have used the free BioRender service, they can use the instructions provided in the link above to download a high-resolution version suitable for publication.
The link provided should only be used for the purposes of this submission. Authors will be charged for figures created on this account if they are not related to this manuscript submission.

LANGUAGE EDITING AND SUPPORT FOR PUBLICATION: If you would like help with English language editing, or other article preparation support, Wiley Editing Services offers expert help, including English Language Editing, as well as translation, manuscript formatting, and figure formatting at www.wileyauthors.com/eeo/preparation. You can also find resources for Preparing Your Article for general guidance about writing and preparing your manuscript at www.wileyauthors.com/eeo/prepresources.

RIGOUR AND REPRODUCIBILITY: We recommend authors consult The Journal's Rigour and Reproducibility requirements to ensure that their manuscript is suitable for publication: https://physoc.onlinelibrary.wiley.com/pb-assets/hub-assets/physoc/documents/TJP-Rigour-and-Reproducibility-Requirements.pdf

REVISION CHECKLIST:
Check that your Methods section conforms to journal policy: https://jp.msubmit.net/cgi-bin/main.plex?form_type=display_requirements#methods

Check that data presented conforms to the statistics policy: https://jp.msubmit.net/cgi-bin/main.plex?form_type=display_requirements#statistics

Upload a full Response to Referees file. To create your 'Response to Referees' copy all the reports, including any comments from the Senior and Reviewing Editors, into a Microsoft Word, or similar, file and respond to each point, using font or background colour to distinguish comments and responses and upload as the required file type.

Please upload two versions of your manuscript text: one with all relevant changes highlighted and one clean version with no changes tracked. The manuscript file should include all tables and figure legends, but each figure/graph should be uploaded as separate, high-resolution files. The journal is now integrated with Wiley's Image Checking service. For further details, see: https://www.wiley.com/en-us/network/publishing/research-publishing/trending-stories/upholding-image-integrity-wileys-image-screening-service

You may also upload:
- 'Potential Cover Art' for consideration as the issue's cover image
- Appropriate Supporting Information (Video, audio or data set: see https://jp.msubmit.net/cgi-bin/main.plex?form_type=display_requirements#supp).

We look forward to receiving your revised submission.

If you have any queries, please reply to this email and we will be pleased to advise.

Yours sincerely,

Bjorn Knollmann
Senior Editor
The Journal of Physiology

REQUIRED ITEMS

(1) You must start the Methods section with a paragraph headed Ethical approval (https://jp.msubmit.net/cgi-bin/main.plex?form_type=display_requirements#methods).

Research must comply with The Journal's policies regarding animal experiments (https://physoc.onlinelibrary.wiley.com/hub/animal-experiments) and adherence to these policies must be stated in the manuscript.

Authors should confirm in their Methods section that their experiments were carried out according to the guidelines laid down by their institution's animal welfare committee, including an ethics approval reference number. The Methods section must contain a statement about access to food, water and housing, details of the anaesthetic regime: anaesthetic used, dose and route of administration, and method of killing the experimental animals.

(2) A Data Availability Statement is required for all papers reporting original data. This must be in the Additional Information section of the manuscript itself. It must have the paragraph heading 'Data Availability Statement'. All data supporting the results in the paper must be either: in the paper itself; uploaded as Supporting Information for Online Publication; or archived in an appropriate public repository. The statement needs to describe the availability or the absence of shared data. Authors must include in their statement: a link to the repository they have used, or a statement that it is available as Supporting Information; reference the data in the appropriate sections(s) of their manuscript; and cite the data they have shared in the References section. Whenever possible, the scripts and other artefacts used to generate the analyses presented in the paper should also be publicly archived. If sharing data compromises ethical standards or legal requirements then authors are not expected to share it, but must note this in their statement. For more information, see our Statistics Policy.

EDITOR COMMENTS

Reviewing Editor:

I thank the authors for presenting a revised version of the manuscript and addressing the questions. The referees indicated that the manuscript was revised adequately, and their comments were addressed. I agree with the referees' assessments and have no additional comments. To fully comply with the journal's guidelines, please indicate the source of the animals in the text, as it only mentions how the KO animals were generated. Moreover, please describe the method of euthanasia used at the end of experiments for both WT animals and KO animals that did not die prematurely.

Please see 'Required Items' above.

Senior Editor:

The manuscript is acceptable after the authors provide the remaining animal ethics issues, and include a data availability statement.

REFEREE COMMENTS

Referee #1:

The authors have thoughtfully and seriously addressed reviewer comments enhancing the manuscript and providing new analyses that also enhance this work. I have no further suggested changes.

Referee #2:

I thank the authors for adequately addressing my comments. The changes made to the text improved the quality of the manuscript, and I have no further comments.

END OF COMMENTS

We thank the reviewers for their thoughtful evaluation of our manuscript. Below, we provided a point-by-point response to each comment in blue. The corresponding changes in the text are highlighted in yellow.

EDITOR COMMENTS

Reviewing Editor:

I thank the authors for presenting a revised version of the manuscript and addressing the questions. The referees indicated that the manuscript was revised adequately, and their comments were addressed. I agree with the referees' assessments and have no additional comments. To fully comply with the journal's guidelines, please indicate the source of the animals in the text, as it only mentions how the KO animals were generated. Moreover, please describe the method of euthanasia used at the end of experiments for both WT animals and KO animals that did not die prematurely.

We have made the requested changes in section 2.2 of the Methods to explain the source of the animals as well as the method of euthanasia used at the end of the experiments.

Senior Editor:

The manuscript is acceptable after the authors provide the remaining animal ethics issues, and include a data availability statement.

We have added the requested animal ethics topics in section 2.2 of the Methods and have moved our data availability statement to Section 5: Additional Information.

REFEREE COMMENTS

Referee #1:

The authors have thoughtfully and seriously addressed reviewer comments enhancing the manuscript and providing new analyses that also enhance this work. I have no further suggested changes.

We appreciate the complimentary comments on our revised manuscript!

Referee #2:

I thank the authors for adequately addressing my comments. The changes made to the text improved the quality of the manuscript, and I have no further comments.

We are pleased to hear that our revisions have addressed each comment satisfactorily.

2nd Revision - Editorial Decision 12-Dec-2024

Dear Dr Glasscock,

Re: JP-RP-2024-287582R2 "Sex-specific differences in mortality and neurocardiac interactions in the Kv1.1 knockout mouse model of sudden unexpected death in epilepsy (SUDEP)" by Kelsey Paulhus, Praveen Kumar, Kelly Kneale, Noah Hutson, Nicole M. Gautier-Hall, Deng-Shan Shiau, Megan Watts, Krystle Trosclair, Hemangini A Dhaibar, Paari Dominic, Leonidas Iasemidis, and Edward Glasscock

We are pleased to tell you that your paper has been accepted for publication in The Journal of Physiology.

The last Word (or similar) version of the manuscript provided will be used by the Production Editor to prepare your proof. When this is ready you will receive an email containing a link to Wiley's Online Proofing System. The proof should be thoroughly checked and corrected as promptly as possible.

Authors should note that it is too late at this point to offer corrections prior to proofreading. Major corrections at proof stage, such as changes to figures, will be referred to the Editors for approval before they can be incorporated. Only minor changes, such as to style and consistency, should be made at proof stage. Changes that need to be made after proof stage will usually require a formal correction notice.

All queries at proof stage should be sent to: TJP@wiley.com.

Following copyediting and typesetting the accepted version will be published online.

Yours sincerely,

Bjorn Knollmann
Senior Editor
The Journal of Physiology

IMPORTANT POINTS TO NOTE FOLLOWING YOUR PAPER'S ACCEPTANCE:

If you would like to receive our 'Research Roundup', a monthly newsletter highlighting the cutting-edge research published in The Physiological Society's family of journals (The Journal of Physiology, Experimental Physiology, Physiological Reports, The Journal of Nutritional Physiology and The Journal of Precision Medicine: Health and Disease), please click this link, fill in your name and email address and select 'Research Roundup':
https://www.physoc.org/journals-and-media/membernews

• TRANSPARENT PEER REVIEW POLICY: To improve the transparency of its peer review process, The Journal of Physiology publishes online as supporting information the peer review history of all articles accepted for publication. Readers will have access to decision letters, including Editors' comments and referee reports, for each version of the manuscript as well as any author responses to peer review comments. Referees can decide whether or not they wish to be named on the peer review history document.

• You can help your research get the attention it deserves! Check out Wiley's free Promotion Guide for best-practice recommendations for promoting your work at: www.wileyauthors.com/eeo/guide. You can learn more about Wiley Editing Services which offers professional video, design, and writing services to create shareable video abstracts, infographics, conference posters, lay summaries, and research news stories for your research at: www.wileyauthors.com/eeo/promotion.

• IMPORTANT NOTICE ABOUT OPEN ACCESS: To assist authors whose funding agencies mandate public access to published research findings sooner than 12 months after publication, The Journal of Physiology allows authors to pay an Open Access (OA) fee to have their papers made freely available immediately on publication.

The Corresponding Author will receive an email from Wiley with details on how to register or log-in to Wiley Authors Services where you will be able to place an order.

• You can check if your funder or institution has a Wiley Open Access Account here: https://authorservices.wiley.com/author-resources/Journal-Authors/licensing-and-open-access/open-access/author-compliance-tool.html.

EDITOR COMMENTS

Reviewing Editor:

I thank the authors for providing the requested information. Congratulations on this publication.

Senior Editor:

This manuscript is now acceptable for publication. Thank you for your excellent contribution to the Journal!
